# Inference of differential gene regulatory networks using boosted differential trees

**DOI:** 10.1093/bioadv/vbae034

**Published:** 2024-02-29

**Authors:** Gihanna Galindez, Markus List, Jan Baumbach, Uwe Völker, Ulrike Mäder, David B Blumenthal, Tim Kacprowski

**Affiliations:** Division Data Science in Biomedicine, Peter L. Reichertz Institute for Medical Informatics of Technische Universität Braunschweig and Hannover Medical School, Braunschweig, 38106, Germany; Braunschweig Integrated Centre of Systems Biology (BRICS), TU Braunschweig, Braunschweig, 38106, Germany; Experimental Bioinformatics, TUM School of Life Sciences, Technical University of Munich, Munich, 85354, Germany; Institute for Computational Systems Biology, University of Hamburg, Hamburg, 22607, Germany; Computational Biomedicine Lab, Department of Mathematics and Computer Science, University of Southern Denmark, Odense, 5230, Denmark; Department of Functional Genomics, Interfaculty Institute for Genetics and Functional Genomics, University Medicine Greifswald, Greifswald, 17475, Germany; Department of Functional Genomics, Interfaculty Institute for Genetics and Functional Genomics, University Medicine Greifswald, Greifswald, 17475, Germany; Biomedical Network Science Lab, Department of Artificial Intelligence in Biomedical Engineering, Friedrich-Alexander-Universität Erlangen-Nürnberg, Erlangen, 91052, Germany; Division Data Science in Biomedicine, Peter L. Reichertz Institute for Medical Informatics of Technische Universität Braunschweig and Hannover Medical School, Braunschweig, 38106, Germany; Braunschweig Integrated Centre of Systems Biology (BRICS), TU Braunschweig, Braunschweig, 38106, Germany

## Abstract

**Summary:**

Diseases can be caused by molecular perturbations that induce specific changes in regulatory interactions and their coordinated expression, also referred to as network rewiring. However, the detection of complex changes in regulatory connections remains a challenging task and would benefit from the development of novel nonparametric approaches. We develop a new ensemble method called BoostDiff (boosted differential regression trees) to infer a differential network discriminating between two conditions. BoostDiff builds an adaptively boosted (AdaBoost) ensemble of differential trees with respect to a target condition. To build the differential trees, we propose differential variance improvement as a novel splitting criterion. Variable importance measures derived from the resulting models are used to reflect changes in gene expression predictability and to build the output differential networks. BoostDiff outperforms existing differential network methods on simulated data evaluated in four different complexity settings. We then demonstrate the power of our approach when applied to real transcriptomics data in COVID-19, Crohn’s disease, breast cancer, prostate adenocarcinoma, and stress response in *Bacillus subtilis*. BoostDiff identifies context-specific networks that are enriched with genes of known disease-relevant pathways and complements standard differential expression analyses.

**Availability and implementation:**

BoostDiff is available at https://github.com/scibiome/boostdiff_inference.

## 1 Introduction

Gene regulation is a fundamental biological process that underlies various cellular functions, including developmental, environmental, and disease contexts. The regulatory relationships in a biological sample can be represented by gene regulatory networks (GRNs), where two gene nodes with a regulatory relationship are connected by an edge (Karlebach and Shamir 2008). GRN inference remains a challenging task because of the inherent complexity of transcriptional regulation, as well as the high dimensionality and noise in biological datasets. Furthermore, GRNs are dynamic and context-specific (Ideker and Krogan 2012, Nagy-Staron *et al.* 2021), i.e. some regulatory processes are active only in certain cell types, tissues, conditions, or in response to specific stimuli. Changes in these pairwise dependencies have been associated with the development of complex diseases (de la Fuente 2010). Differential network analysis, which aims to detect altered connectivity between different conditions or disease states, has recently emerged as a powerful complement to standard differential expression (DE) analysis and is more suitable for detecting context-specific GRNs (de la Fuente 2010, Marbach *et al.* 2012). Exploring how GRN structures are rewired between two different states can reveal molecular mechanisms that drive disease development and progression and identify more relevant therapeutic targets.

Various approaches for deriving differential networks have been the focus of recent studies (Bhuva *et al.* 2019, Basha *et al.* 2020, Baur *et al.* 2020). Representative methods are shown in Table 1. The *z*-score method performs Fisher transformation of Pearson’s correlation coefficients between two conditions. The resulting *z*-scores are modeled as a normal distribution, followed by a *z*-test to detect significant pairwise edges (Zhang *et al.* 2007). Diffcoex, a framework based on weighted gene co-expression analysis (WGGNA), first builds a correlation matrix for each condition being compared and then calculates their difference. From the difference matrix, a dissimilarity matrix is calculated based on a topological overlap measure and hierarchical clustering is applied to find the differentially co-expressed gene clusters (Tesson *et al.* 2010). Another approach, a Gaussian graphical model (GGM)-based method, learns the differential network from conditional associations (Chu *et al.* 2011). EBcoexpress relies on empirical Bayes’ estimation to calculate the posterior probability that an edge is differentially co-expressed (Dawson and Kendziorski 2012, Dawson *et al.* 2012).

**Table 1.** Overview of methods used to derive differential networks and patient-specific networks.^a^

Method	Algorithmic approach	Test	Directionality	No. of conditions	Network type	Reference
BoostDiff	Tree-based		Yes	Two	Differential network	This paper
*z*-score	Correlation-based	*z*-test	No	Two	Differential network	Zhang *et al*. (2007)
EBcoexpress	Empirical Bayes; correlation		No	Multiple	Differential network	Dawson and Kendziorski, (2012)
Diffcoex	Correlation-based	Permutation test	No	Multiple	Differential network	Tesson *et al*. (2010)
GGM-based	GGM; posterior odds	Permutation test	No	Two	Differential network	Chu *et al*. (2011)
chNet	GGM; differential expression	t-test	No	Two	Differential network	Tu *et al*. (2021)
LIONESS	Linear interpolation		No		Patient-specific	Kuijjer *et al*. (2019)
Tanaka *et al.* (2020)	Nonparametric Bayesian network; Δ Edge contribution value	*t*-test	Yes		Patient-specific	Tanaka *et al*. (2020)
DEVC-net	Differential expression variance covariance (DEVC)	*t*-test; Wilcoxon rank sum test	No	Two	Differential network; patient-specific	Yu *et al.* (2015)

a Adapted from Bhuva *et al*. (2019).

The differential network methods described above measure linear relationships or rely on joint normality assumptions, which may not hold in practice (Shojaie 2021). Nonlinear regulatory dependencies are common mechanisms that occur in biology (Brunel *et al.* 2010, Costello *et al.* 2014) and these complex relationships from real datasets may be difficult to detect using correlation- or GGM-based methods. As discussed in a recent review, new methods for differential network analysis for non-Gaussian data are needed (Shojaie 2021). Some methods for inferring patient-specific networks have also been developed. To measure the sample-specific edge contributions, DEVC-net (Yu *et al.* 2015) uses a method that calculates an integrated score called DEVC that considers the differential expression of a gene, differential expression variance of a gene, and covariance of gene pairs. This method uses a template network to identify differential network features from gene expression data and can be used to obtain sample-specific networks. Linear Interpolation to Obtain Network Estimates for Single Samples (LIONESS) reverse engineers sample-specific networks from an aggregate network by applying linear interpolation on the edge weights from the aggregate networks (Kuijjer *et al.* 2019). This approach can be used in combination with existing network inference methods to also capture nonlinear dependencies. Tanaka *et al.* (2020) infer nonparametric Bayesian networks using B-spline regression. From the model parameters, the method derives a value representing the contribution of each edge to the expression in a particular sample, called the edge contribution value (ECv). The absolute differences in ECvs (ΔECv) between certain samples are then used to build sample-specific networks. An alternative to the abovementioned approaches are tree-based strategies, which offer the advantage of more relaxed model assumptions. While examples such as GENIE3 and derived tools continue to be successfully applied in various biological settings (Huynh-Thu *et al.* 2010, Moerman *et al.* 2019, Van de Sande *et al.* 2020), they cannot be used to directly compare two different biological conditions.

**Figure 1.**
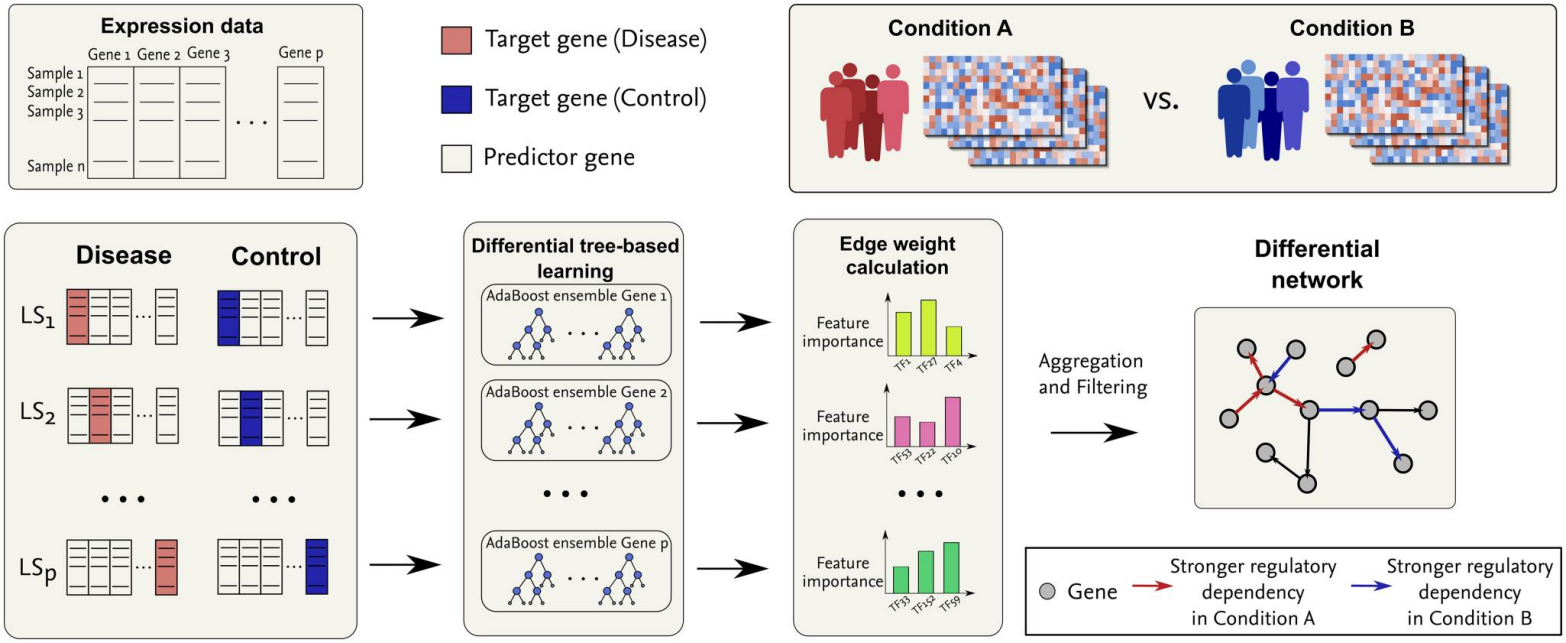
Overview of the BoostDiff algorithm. As input, we require two gene expression matrices corresponding to a target condition (e.g. disease) and a baseline condition (e.g. control). For each of p total genes, a learning subsample (LS) is drawn from the two datasets, after which an AdaBoost ensemble of differential trees is built to identify the features that are more predictive of the gene expression levels in the target condition. By setting a target condition, BoostDiff can be used to identify regulatory relationships that are more pronounced in condition A (e.g. disease state) and condition B (e.g. control/healthy), thereby providing a differential network capturing context-specific regulatory changes. In the overall workflow, the BoostDiff algorithm is run twice, once with condition A as target condition and subsequently with B as target condition. The results are then combined to obtain the final differential network. Most notably, while existing approaches aim for the reconstruction of whole genome-scale GRNs, BoostDiff concentrates on maximizing the precision for those parts of the regulatory network that actually characterize the difference between the two phenotypes.

We introduce BoostDiff, a nonparametric tree-based approach for reconstructing directed differential networks (Fig. 1). The main component of BoostDiff is the differential tree, a modified version of the standard regression tree that can be used to identify gene pairs exhibiting changes in regulatory dependencies between two biological conditions. To build the differential trees, we use a novel splitting criterion called the differential variance improvement (DVI), which measures the difference in predictive value of a feature on the gene expression levels between two conditions. We demonstrate that boosting the differential trees with respect to samples belonging to a specific target condition is an important step for promoting condition specificity of the output networks. Tree-based variable importance measures are then calculated to obtain a ranking of regulators and to derive the differential network. Using simulated gene expression data, we evaluate the performance of BoostDiff in comparison to baseline methods and existing differential network tools. We further apply BoostDiff to multiple transcriptomics datasets, including COVID-19, Crohn’s disease, prostate adenocarcinoma, and breast cancer data, as well as stress response data in the bacterium *Bacillus subtilis*.

## 2 Methods

### 2.1 Overview of the differential network inference approach

The overall strategy of BoostDiff is inspired by the popular network inference tool GENIE3 (Huynh-Thu *et al.* 2010). GENIE3 aims to reconstruct a network using as input a gene expression matrix derived from a single biological condition. For this, GENIE3 fits an ensemble of regression trees, using the expression levels of the target genes as the output variable and the expression levels of potential regulators as the features. The splitting criterion for a node in the regression tree is the reduction

$$\begin{array}{c} |S|\cdot \text{Var}(S)\;-\;|S_{L}|\cdot \text{Var}(S_{L})\;-\;|S_{R}|\cdot \text{Var}(S_{R}), \end{array}$$

in variance (impurity) of the target gene’s expression caused by the split. Here, *S* denotes the set of samples that reach a node to be split, $S_{L}$ and $S_{R}$ are the subsets of samples in the left and right branches of the split, respectively, and $\text{Var}(S^{\prime })$ is the variance of the output variable in a set of samples $S^{\prime }\in \{S,S_{L},S_{R}\}$. The importance of one variable in a single regression tree is calculated by summing up the reductions in variance of all tree nodes where this variable is used as a splitting feature. The overall importance of a feature in the ensemble is then averaged across all trees. One ensemble model is learned for each target gene in the dataset. The tree-based feature importances based on variance reduction are interpreted as edge weights of candidate regulator-target gene links. A high edge weight indicates that a regulator has a large contribution in predicting the expression level of a target gene. The edges with the highest weights are returned as the inferred GRN. Biologically, the top-ranked edges inferred by GENIE3 represent the relationships that are dominant in the condition of interest.

Similarly, the differential network inference problem can be decomposed into *p* independent regression subproblems, where *p* is the total number of genes in the expression data. Our strategy assumes that, in a given biological context, the expression level of a gene can be modeled as a function of the expression levels of other genes (Fig. 1). The crucial difference between BoostDiff and GENIE3 is that we simultaneously take into account two datasets for inferring a differential network. More precisely, our approach requires the availability of (1) gene expression data matrix

$$\begin{array}{c} X^{D}={\left(x_{i,g}^{D}\right)}_{\left(i,g)=(1,1\right)}^{\left(N_{D},p\right)} \end{array}$$

for $N_{D}$ measurements from a disease condition and (2) the matrix

$$\begin{array}{c} X^{C}={\left(x_{i,g}^{C}\right)}_{\left(i,g)=(1,1\right)}^{\left(N_{C},p\right)} \end{array}$$

for $N_{C}$ measurements from a control condition. Both matrices have *p* columns (total number of genes). That is, $x_{i,g}^{D}$ contains the expression of gene *g* in measurement (patient) *i* from the disease condition.

The inference task is modeled as a feature selection problem that aims to find features that are more predictive of expression levels in a target condition than in the baseline condition. For this, differential network analysis is performed by solving the regression problem while taking into account information from two distinct labels. We use the AdaBoost algorithm (Drucker 1997) using differential trees as base learners to drive the improved prediction of expression levels in the target condition. The trained model provides a ranking of the edges by computing a feature importance weight for each regulator (see details below).

For each target gene g=1,…,p, we define regression problems $LS^{D}(g)=(X_{-g}^{D},X_{•,g}^{D})$ and $LS^{C}(g)=(X_{-g}^{C},X_{•,g}^{C})$. The design matrices $X_{-g}^{D}$ and $X_{-g}^{C}$ are obtained by deleting the *g*th columns $X_{•,g}^{D}$ and $X_{•,g}^{C}$ from $X^{D}$ and $X^{C}$, respectively. The target variables are set to the deleted columns $X_{•,g}^{D}$ and $X_{•,g}^{C}$. The inference of our differential network is then performed as follows:

1. For each target gene g=1,…,p:
  - Generate the design matrices and target variable vectors for the regression problems $LS^{D}(g)$ and $LS^{C}(g)$ for gene *g* for the disease and the control conditions.
  - Train an ensemble of differential regression trees with the disease selected as target condition, i.e. tree ensembles that yields a good fit for $LS^{D}(g)$ but not for $LS^{C}(g)$.
  - From the ensemble of differential trees, extract weights for all potential regulators of *g* which are large if the expression of the regulator is highly predictive of *g* in the disease condition but not in the control condition.
2. Aggregate and sort the *p* gene rankings to obtain a global ranking of all differential regulatory edges.
3. Repeat the steps 1 and 2 with reversed roles of *D* and *C* to identify regulatory links that are present in the control but not in the disease condition.

In the following, we describe the details of the algorithm.

### 2.2 Step 1a: Growing a differential tree

We first explain how to build a single differential tree, the key ingredient of step 1a of the algorithm outlined above. We describe how to build the differential tree for the case where the disease has been selected as the target condition. The workflow with control as target condition is analogous.

A differential tree is built through binary recursive partitioning. The key difference to standard regression trees is that, to determine the features (i.e. genes) used for splitting the samples at the inner nodes of our trees, we use a novel split criterion called differential variance improvement (DVI) instead of variance reduction. At each node of the differential tree, we maintain subsets $S^{D}\subseteq \{1,\ldots ,N_{D}\}$ and $S^{C}\subseteq \{1,\ldots ,N_{C}\}$ of the disease and control samples, respectively. Given a possible split feature (i.e. candidate predictor gene) $g^{\prime }$, we define $\text{DVI}(g^{\prime })$ as follows:

$$\begin{array}{c} \text{DVI}(g^{\prime })=\underset{\tau }{\max}\text{VarRed}(g^{\prime },\tau ,S^{D})\\ -\underset{\tau }{\max}\text{VarRed}(g^{\prime },\tau ,S^{C}) \end{array}$$

For a fixed $g^{\prime }$ and splitting threshold τ, the variance reduction for the disease samples is given by

$$\begin{array}{c} \text{VarRed}(g^{\prime },\tau ,S^{D})=\text{MSE}(X_{•,g}^{D}[S^{D}])\\ -\frac{\left|S_{L}^{D}(g^{\prime },\tau )\right|}{\left|S^{D}\right|}\cdot \text{MSE}(X_{•,g}^{D}[S_{L}^{D}(g^{\prime },\tau )])\\ -\frac{\left|S_{R}^{D}(g^{\prime },\tau )\right|}{\left|S^{D}\right|}\cdot \text{MSE}(X_{•,g}^{D}[S_{R}^{D}(g^{\prime },\tau )]), \end{array}$$

where MSE(·) is the mean squared error from the sample mean, $X_{•,g}^{D}[S]=\{x_{i,g}^{D}|i\in S\}$ is the restriction of the target variable to the disease samples (rows) contained in a set of samples *S*, and $S_{L}^{D}(g^{\prime },\tau )=\{i\in S^{D}|x_{i,g^{\prime }}^{D}\leq \tau \}$, and $S_{R}^{D}(g^{\prime },\tau )=\{i\in S^{D}|x_{i,g^{\prime }}^{D}>\tau \}$ are the subsets of disease samples $S^{D}$ available at the current node whose split gene expression levels are, respectively, smaller or larger than the threshold τ. Variance reduction for the control samples is defined analogously. A positive value of the DVI hence means that the gene $g^{\prime }$ is more predictive of *g’*s expression level in the disease condition than in the control condition, whereas a negative DVI value indicates that the opposite is the case.

When building our differential trees, we use genes $g^{*}$ maximizing $\text{DVI}(g^{\prime })$ as split features (see details below). If disease has been selected as the target condition, these genes correspond to regulators of *g* which are much more active in disease samples than in control samples. If control has been selected as the target condition, they correspond to regulators in healthy individuals that have lost their regulatory activity in the disease.

Given the regression problems $LS^{D}(g)$ and $LS^{C}(g)$ for the disease and control conditions, respectively, we construct a differential regression tree whose nodes are 5-tuples

$$\begin{array}{c} v=(S^{D},S^{C},g^{*},\tau_{D}^{*},\tau_{C}^{*}), \end{array}$$

where $g^{*}$ is the split gene, $\tau_{D}^{*}$ is the split threshold for the disease samples, and $\tau_{C}^{*}$ is the split threshold for the control samples. Note that we use two different thresholds, since using a single threshold for both conditions while optimizing the DVI leads to a skewed expression distribution in each side of the split, with one side favoring disease samples and the other side favoring control samples. The construction is done as follows:

1. Initialize root as
  $$\begin{array}{c} r=(S^{D}=\{1,\ldots ,N_{D}\},S^{C}=\{1,\ldots ,N_{C}\},•,•,•), \end{array}$$
  where ‘•’ is a placeholder for not yet defined split genes and thresholds.
2. Starting at the root, recursively construct a differential tree via binary partitioning as follows:
3. At the current node $v=(S^{D},S^{C},•,•,•)$ of the tree under construction, do the following:
  - If a suitable termination criterion (the maximum depth of the tree has been reached or the number of target or baseline samples reaching in a node has been reached) has been satisfied or $\max_{g^{\prime }}\text{DVI}(g^{\prime })\leq 0$, label *v* as leaf and traceback.
  - Otherwise, set the node *v’*s split gene to
    $$\begin{array}{c} g^{*}=\underset{g^{\prime }}{\text{argmax}}\text{DVI}(g^{\prime }), \end{array}$$
    its disease threshold to
    $$\begin{array}{c} \tau_{D}^{*}=\underset{\tau }{\text{argmax}}\text{VarRed}(g^{*},\tau ,S^{D}), \end{array}$$
    and its control threshold to
    $$\begin{array}{c} \tau_{C}^{*}=\underset{\tau }{\text{argmax}}\text{VarRed}(g^{*},\tau ,S^{C}). \end{array}$$
  - Initialize *v’*s left child as
    $$\begin{array}{c} v_{L}=(S_{L}^{D}(g^{*},\tau_{D}^{*}),S_{L}^{C}(g^{*},\tau_{C}^{*}),•,•,•), \end{array}$$
    its right child as
    $$\begin{array}{c} v_{R}=(S_{R}^{D}(g^{*},\tau_{D}^{*}),S_{R}^{C}(g^{*},\tau_{C}^{*}),•,•,•), \end{array}$$
    and continue with processing $v_{L}$ and $v_{R}$.

In the regression trees described by (Breiman *et al.* 1984), the prediction for a sample is determined by traversing the tree until a leaf node is reached. Here, we are more interested in predicting the expression values of the samples in the target condition; thus, prediction is performed only for target samples using the identified splitting thresholds $\tau_{D}^{*}$. The final prediction is calculated as the expected value of the expression levels of the target samples assigned to the leaf nodes after fitting the differential tree.

### 2.3 Step 1b: Boosting the differential trees

Inspired by GRNBoost2 (Moerman *et al.* 2019), we implement a boosting algorithm that derives a strong prediction model by sequentially training a pool of differential trees as the weak learners. AdaBoost for regression is typically used for solving problems where the output is a continuous variable (i.e. expression levels) without explicitly considering the class of the samples. Here, we adapted the AdaBoost.R2 algorithm (Drucker 1997) to handle the regression problem, given labels from two classes (i.e. conditions). Using the differential trees as base learners, the modified algorithm performs the boosting with respect to samples belonging to the specified target condition. In this way, BoostDiff attempts to find a model that is more predictive of the target condition compared to the baseline condition. In each tree, only the target samples are re-weighted in subsequent boosting iterations, while samples from the baseline condition retain uniform weight. In particular, target samples that are more difficult to predict are selected with higher weights during the bootstrapping step and will always be compared to a uniform sample from the baseline condition. To avoid overfitting, we set a low number of trees and in practice find that 50–100 differential trees in the ensemble are sufficient for real transcriptomics datasets. The algorithm is described in detail in the Supplementary Methods section.

### 2.4 Step 1c: Computing variable importances to rank the candidate predictors

Tree-based methods allow for the calculation of a variable importance measure that can be used to rank the features according to their relevance for predicting the output. In GENIE3, the importance of a predictor gene $g^{\prime }$ is calculated as the sum of the variance reduction across all nodes where $g^{\prime }$ is used as the splitting feature, averaged over all trees in the ensemble. For BoostDiff, we derive a similar measure by considering the samples belonging to the target condition (step 1c in the high-level description of BoostDiff given above). If disease is selected as target condition, the importance $\text{VIM}(g^{\prime })$ attributed to a candidate predictor gene $g^{\prime }$ is calculated as the weighted variance reduction for the disease samples across all *M* trees in the ensemble:

$$\begin{array}{c} \text{VIM}(g^{\prime })=\sum_{m=1}^{M}\alpha^{m}\cdot \sum_{v\in V_{m}(g^{\prime })}\text{VarRed}(g^{\prime },\tau_{v}^{D},S_{v}^{D}) \end{array}$$

Here, *m* is the boosting iteration, $\alpha^{m}$ is the weight of the differential tree returned by AdaBoost, $V_{m}(g^{\prime })$ is the set of nodes in tree *m* where $g^{\prime }$ was used as the split feature, and $\text{VarRed}(g^{\prime },\tau_{v}^{D},S_{v}^{D})$ is the variance reduction for the disease samples at a node $v=(S_{v}^{D},•,g^{\prime },\tau_{v^{\prime }}^{D},•)\in V_{m}(g^{\prime })$. Since each node in a differential tree has two independent thresholds, interpreting the tree becomes more abstract with increasing depth. Boosting using shallow differential trees (e.g. differential tree stumps) thus favors greater interpretability of the variable importance measure.

### 2.5 Steps 2 and 3: Edge ranking and filtering from boosted differential trees

Each modified AdaBoost model yields a separate ranking of the regulators. However, simply ordering the regulatory links according to the weights leads to a bias toward highly variable target genes. To avoid this, we first scale the expression levels of each target gene to unit variance, similarly implemented in GENIE3 (Huynh-Thu *et al.* 2010).

Boosting with respect to a target condition does not necessarily produce a model that predicts a gene’s expression in the target condition better than its expression in the baseline condition. To illustrate, sample plots of the training progression are shown in Supplementary Fig. S1. To restrict the results to differential edges, we recommend examining the distributions of the mean difference in prediction error. Sample distributions of these values from the simulated and real transcriptomics data are shown in Supplementary Figs S2 and S3, respectively. Based on these generated plots, users can filter for target genes with lower mean prediction error in the target condition than the baseline condition by applying a threshold. Alternatively, users can select the top edges with the lowest mean difference in prediction error or input a user-defined percentile. After filtering, the edges are re-ranked based on the variable importance measure used as the edge weight. The procedure is performed for each of the two sub-analyses (disease or control as target condition). The top edges (user-specified parameter) from each sub-analysis are then merged to obtain the final network.

Given the ensemble nature of BoostDiff where each differential tree is constructed differently from randomly selected predictors, deriving an explanation of the model’s predictions is challenging. To obtain more mechanistic and biologically meaningful results, we can rely on the topology of the differential network and use community detection algorithms that aim to find highly connected subnetworks that could be potentially associated with the disease of interest.

### 2.6 Compared methods

We evaluate the performance of BoostDiff against baseline methods and existing differential networks methods. To verify the condition specificity of the networks output by BoostDiff, we compare its performance to those of two GRN inference methods, GENIE3 and ARACNE (Margolin *et al.* 2006). GENIE3 is run using the corresponding R package. ARACNE is run using the implementation provided in the R package *minet* (Meyer *et al.* 2008). For both GENIE3 and ARACNE, only the gene expression matrix from the disease condition is used as input. We also compare BoostDiff to two additional baseline methods: (i) random forests of differential trees (‘RF Difftrees’) and (ii) a ‘Differential GENIE3’ approach, where we separately infer the networks from each condition using GENIE3 and subtract the calculated edge weights derived from the control condition from the weights derived from the disease condition and vice versa. For details, an AIMe report is available at https://aime.report/656I3Z/2 (Matschinske *et al.* 2021).

Next, we compare the performance of BoostDiff to other differential network methods from the benchmarking study in Bhuva *et al.* (2019). These methods include *z*-score, EBcoexpress, Diffcoex, and a GGM-based method, which are implemented in the *dcanr* package in R. Additionally, we run the chNet algorithm (Tu *et al.* 2021), which considers significant changes in both partial correlations of edges and differential expression. To facilitate comparability and given that only the tree-based methods provide directionality information among the methods examined here, we convert directed edges to undirected edges (Bhuva *et al.* 2019).

### 2.7 Evaluation using simulated datasets

Gene expression data for disease and control conditions are simulated by adapting the SimulatorGRN approach (Bhuva *et al.* 2019), a dynamical systems modeling tool to simulate differential co-expression patterns. SimulatorGRN induces differential co-expression by perturbing a reference network via gene knockdown to reduce their expression levels and models activation/repression of a gene by a set of regulators using normalized Hill equations. Knockdown leads to complex nonlinear differences between the generated disease and control samples (Supplementary Fig. S4). By default, known regulatory interactions in *Saccharomyces cerevisiae* are used to create a reference GRN. To obtain the ‘true’ differentially co-expressed edges and allow performance evaluation, genes in the network whose abundance are affected by the knockdown perturbation and thus exhibit regulatory dependency are determined via sensitivity analysis.

In the original SimulatorGRN framework, a sample can have multiple genes knocked down, even though the evaluation considers each knockdown gene separately. To explicitly eliminate the confounding effect of additional knockdown genes in our experiments, we generate the expression data in the perturbed condition such that exactly one randomly selected input gene is knocked down. We evaluate the different tools across four scenarios: (i) 50 nodes; 500 simulations, (ii) 150 nodes; 500 simulations, (iii) 300 nodes; 500 simulations, and (iv) 500 nodes; 250 simulations. In each simulation, 100 samples are generated per condition. The final disease samples are those which have the gene knockdown, whereas the control samples are ‘wild type’ (Bhuva *et al.* 2019). The edges from the ‘association network’ of each simulation are used as the ground truth edges for evaluating performance. The parameters for generating the simulated data are shown in Supplementary Table S1.

In the analyses on simulated data, all genes except the target gene are considered as potential regulators. The *z*-score method, chNet, Diffcoex, and the GGM-based method are run with the default parameters. The parameters used for the RF Difftrees and BoostDiff are provided in Supplementary Table S2. For each simulation, we filter for the target genes belonging to the 3rd percentile based on the mean difference in prediction errors. The parameters for the EBcoexpress runs are shown in Supplementary Table S3.

The different tools have different statistical methods and cutoffs for determining the differentially coexpressed edges depending on how the algorithm works. To facilitate comparability, we show the top *k* predicted edges output by each method (except for chNet, wherein the number of predicted differential edges depends on the tuning parameter and is variable for each simulation; thus, extracting the top *k* edges cannot be consistently applied across simulations). For visualization, we show the results based on the top 100 predicted edges output by each method for the simulated data. We report the performance using precision, recall, and F1 score as the evaluation metrics.

### 2.8 Evaluation using real datasets

For evaluation on real datasets, BoostDiff, Diffcoex, EBcoexpress, the *z*-score method, and Differential GENIE3 are run on gene expression data for four human diseases (COVID-19, Crohn’s disease, breast cancer, and prostate adenocarcinoma) and for the stress response in *B.subtilis*. For the human diseases, we extract the top 1000 edges to generate the final network. For the *B.subtilis* data, we use the top 300 edges.

For the COVID-19 RNA-Seq dataset, raw gene counts are downloaded from the Gene Expression Omnibus (GEO) database under the accession number GSE156063 (Mick *et al.* 2020). We use data generated from nasal swab samples from COVID-19 (n=93) and uninfected patients (n=100). Count data are normalized using the DESeq2 package in R with the variance stabilizing transformation (vst}) function. Differentially expressed genes (DEGs) are obtained using DESeq2 (Love *et al.* 2014).

For the Crohn’s disease (CD) dataset, normalized microarray data are downloaded from the GEO database under the accession number GSE126124 (Palmer *et al.* 2019), which were generated from colon biopsies of individuals with CD (n=37) and healthy controls (n=19). Differential expression analysis is performed using limma (Ritchie *et al.* 2015).

To evaluate BoostDiff on data from The Cancer Genome Atlas (TCGA), preprocessed RNA-Seq gene expression data and DEGs from primary tumors and normal solid tissues are obtained from https://xenabrowser.net/ (Goldman *et al.* 2020). We use gene expression data for two cancer types, namely, prostate adenocarcinoma (PRAD) and breast cancer (BRCA), both of which contain at least 50 samples for the normal tissues.

For the human datasets, Illumina IDs are converted to HGNC symbols using the R package biomaRt where applicable (Durinck *et al.* 2009). Expression levels corresponding to probes mapped to the same gene symbol are averaged. The top 20% of genes with the lowest variance are filtered out. To generate the final GRN and facilitate comparability with BoostDiff, we filter the outputs for edges containing a TF, using the list of human transcription factors downloaded from http://humantfs.ccbr.utoronto.ca/ (Lambert *et al.* 2018). These TFs are also used as the candidate regulators for BoostDiff. Genes whose mean difference in prediction error of the models are more extreme than the threshold identified from the 3rd percentiles of the distributions are retained. For the CD dataset, a more stringent filtering step is performed because of the small sample size available for inference. All the outputs from the different methods are filtered for the top 1000 edges (except for chNet). For BoostDiff, the final network is thus comprised of the combined top 500 edges from the run where the disease condition is set as the target condition and the top 500 edges from the run where the control condition is set as the target condition. To identify more specific candidate network modules and facilitate the interpretability of the resulting networks, we apply the Louvain community detection algorithm using the python-louvain package (https://github.com/taynaud/python-louvain). The enrichr module of the gseapy v. 1.1.0 package is used to identify enriched KEGG pathways in the output networks (Subramanian *et al.* 2005, Kuleshov *et al.* 2016). KEGG terms are considered to be significantly enriched in a network if the overlap with the gene set is at least three genes.

We further test the performance of BoostDiff using transcriptome data related to the well-characterized general stress response in *B.subtilis* (Nicolas *et al.* 2012). We examine three contexts, namely, response to salt stress, response to heat stress, and response to ethanol stress, all of which are known to induce the SigB regulon. Expression data for all protein-coding genes and RNA features (S1 to S1583) of *B.subtilis* from the corresponding samples were downloaded from the GEO database (accession number GSE27219). Considering that bacterial adaptation is characterized by smaller variation across samples than in animal experiments or experiments with human specimens, gene expression datasets for bacterial adaptation generally have low number of replicates. Therefore, we generate additional synthetic samples using Synthetic Minority Over-sampling TEchnique (SMOTE) (https://github.com/akazs/pySMOTE/) to obtain 48 samples per condition to be available for inference. We note that a large sample size from experimental data still remains ideal for using BoostDiff; SMOTE is used as a workaround in order to be able to run the compared network inference methods on the *B.subtilis* datasets. Genes with low variance are filtered out using a variance cutoff of 0.4. For the BoostDiff runs, the final network is obtained by filtering the differentially predicted target genes based on the 3rd percentile and combining the top 150 edges in the differential network for each of the two sub-analyses. The results of the compared methods are filtered for the top 300 edges to obtain the differential network. Enrichment analyses for the *B.subtilis* outputs is performed using the ShinyGO v. 0.77 (Ge *et al.* 2020).

## 3 Results and discussion

### 3.1 BoostDiff outperforms existing methods on complex simulated settings

**Figure 2.**
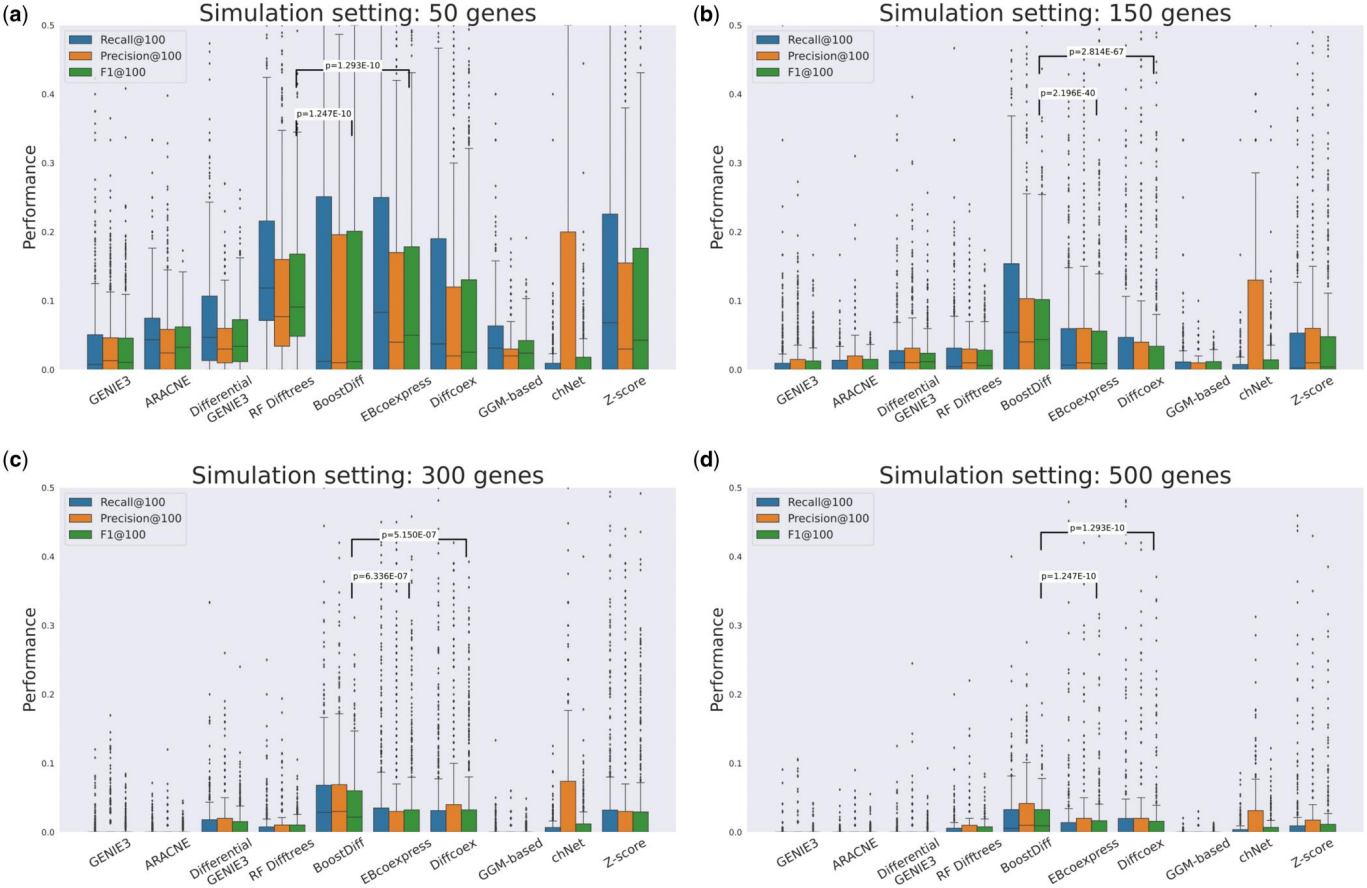
Performance of differential trees and BoostDiff compared to baseline methods and other differential network tools using simulated data comprising (a) 50 genes; (b) 150 genes; (c) 300 genes; (d) 500 genes. For all methods, the top 100 edges were considered (except for chNet). The one-sided Mann–Whitney U test is used to calculate the *P*-values.

As expected, BoostDiff better identifies the differential edges than GENIE3 and ARACNE, both of which infer a static network from the disease condition alone (Fig. 2). The Differential GENIE3 method shows similar performance to those of the baseline methods, despite using the expression data from both disease and control conditions. These findings indicate that the feature importances calculated from the trees built using DVI as the splitting criterion are useful for identifying the differential edges. In addition, in all but the smallest simulation scenarios, the boosting scheme performs significantly better than the random forest of differential trees (see results for BoostDiff and RF Difftrees in Fig. 2): While RF Difftrees outperformed all the other methods in the 50-gene setting, BoostDiff is the best performing methods in the more complex 150-, 300-, and 500-gene settings. Results are similar for varying cutoffs of the number of top edges (k∈{50,100,150,200}) (Supplementary Fig. S5).

We then determine the rank of each method in terms of the F1 score across all simulations in the four simulation settings. The results show that BoostDiff is the most frequently top-ranked method in the majority of simulations, more notably in the simulation settings with larger numbers of genes (Supplementary Fig. S6). In addition, except for when a high number of base learners is used in the ensemble, the performance of BoostDiff is consistent across different hyperparameter combinations (no. of features, maximum tree depth) (Supplementary Fig. S7).

### 3.2 Results on real datasets

#### 3.2.1 COVID-19

**Figure 3.**
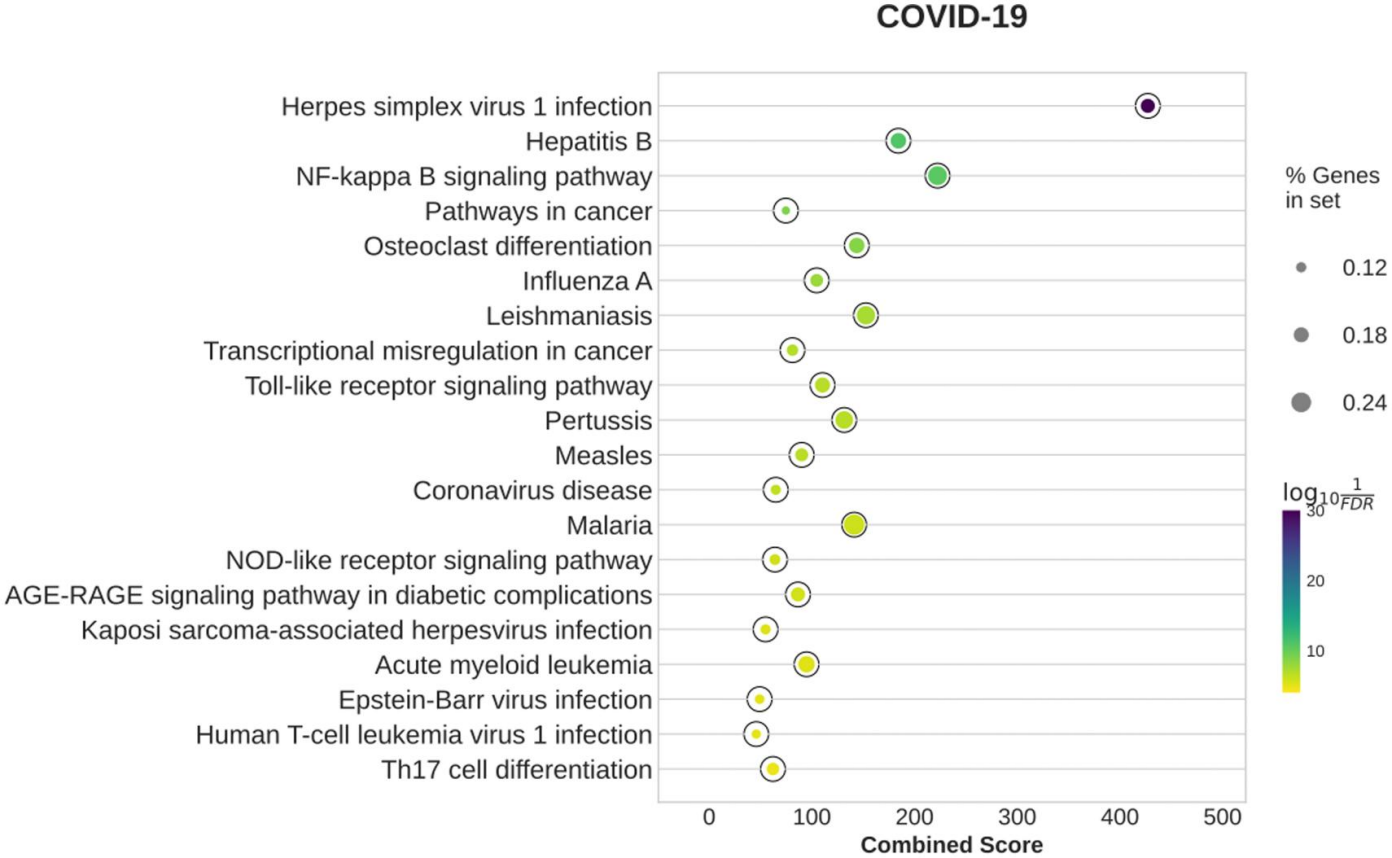
Enriched KEGG pathways in the networks inferred by BoostDiff for the COVID-19 dataset. The resulting differential networks were strongly enriched in inflammation-related and other relevant pathways, consistent with known disease pathophysiology.

The network inferred by BoostDiff shows stronger enrichment of COVID-19-related pathways compared to those of Diffcoex and the *z*-score method, whereas all edges in the EBcoexpress output are assigned zero posterior probabilities (Fig. 3 and Supplementary Tables S4–S7). In particular, the Diffcoex and *z*-score networks are enriched in less specific terms, such as ‘Influenza A,’ ‘Measles,’ and ‘Human T-cell leukemia virus 1 infection.’ Differential GENIE3 returned ‘Coronavirus disease,’ ‘Neutrophil extracellular trap formation,’ and ‘Phagosome.’ In addition to terms related to various pathogenic infections, the BoostDiff network is significantly enriched in COVID-19-relevant pathways, such as ‘Coronavirus disease,’ ‘TNF signaling pathway,’ ‘Th17 cell differentiation pathway,’ ‘NF-κB signaling pathway,’ ‘NOD-like receptor pathway,’ ‘Toll-like receptor signaling pathway,’ and ‘Viral protein interaction with cytokine and cytokine receptor’ (Fig. 3). However, similar to BoostDiff, ‘Herpes simplex I infection’ is a top enriched term in the networks output by both methods, which could indicate the presence of shared genes activated in response to viral infection. For the BoostDiff network, examining the differential edges when considering the two sub-analyses separately shows generally similar results, indicating the enrichment of infection-related pathways (Supplementary Fig. S8). We also compare the BoostDiff network to the list of DEGs. While the overlap between the differential network nodes and DEGs is significant, it is quite low (Jaccard similarity=0.129). Further, removing the DEGs from the genes in the differential network retains the enrichment of COVID-19-related pathways (Supplementary Fig. S9), indicating that these dysregulated genes identified by BoostDiff are missed by standard DE analysis.

**Figure 4.**
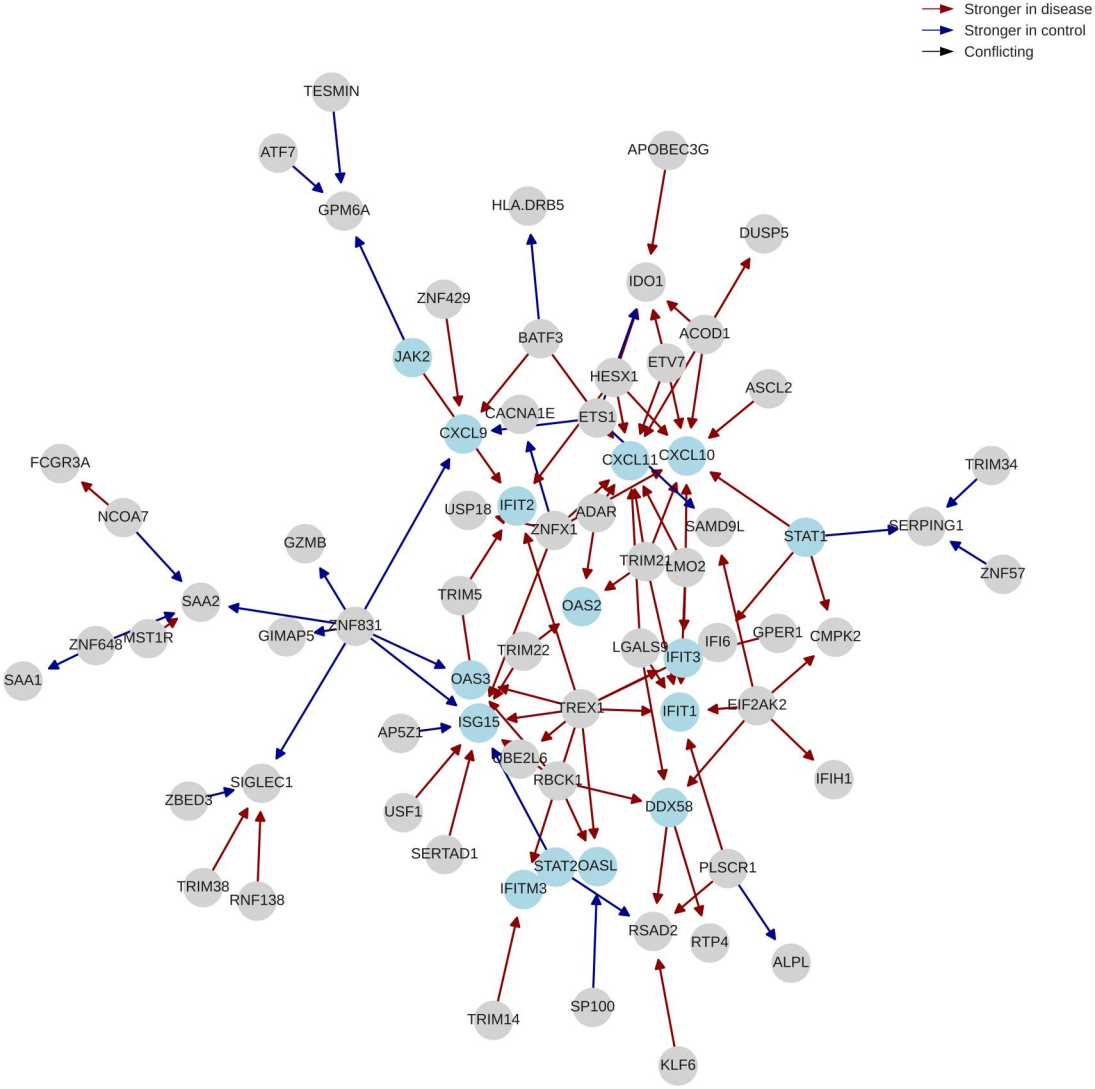
Dysregulated module identified from the COVID-19 differential network inferred by BoostDiff using the Louvain algorithm. Some notable genes in the module include *JAK2, CXCL9, CXCL10, CXCL11, DDX58, STAT1, STAT2, IFIT1, IFIT2, IFIT3, IFITM3, OASL, OAS2, OAS3*, and *ISG15*.

To further examine the differential networks, we apply the Louvain community detection algorithm (Blondel *et al.* 2008). BoostDiff, Diffcoex, *z*-score, and Differential GENIE3, identifies modules that are enriched in viral infection terms such as ‘Influenza A,’ ‘Coronavirus disease,’ ‘Hepatitis C,’ as well as signaling pathways ‘NOD-like receptor signaling pathway,’ ‘RIG-1-like receptor signaling pathway,’ and ‘Toll-like receptor signaling pathway’ (Supplementary Figs S10–S12 and Supplementary Tables S8–S11). However, the BoostDiff module is additionally enriched in the terms ‘Cytosolic DNA-sensing pathway’ and ‘Chemokine signaling pathway’ (Fig. 4 and Supplementary Tables S10 and S11). Notable COVID-19-related genes in the BoostDiff module include *CXCL10, DDX58, STAT1, STAT2, EIF2AK2*, and *ISG15.* Other additionally known genes involved in pathogen response include *IFIT1, IFIT2, IFIT3, CXCL11*, and *CXCL9*. Chemokines are produced in response to a range of viral infections. In COVID-19, chemokine signaling has been linked to acute respiratory distress syndrome (Zhou *et al.* 2020). *DDX58* (RIG-1) is involved in the production of interferons in response to COVID-19 (Yamada *et al.* 2021). Interferon signaling mediated by STAT1 and STAT2 is a key antiviral defense mechanism, and SARS-CoV-2 was demonstrated to inhibit nuclear translocation of STAT1 and STAT2 (Mu *et al.* 2020). OASL, OAS2, and OAS3 are members of the oligoadenylate synthetase family of proteins, which are involved in the antiviral activity of interferons and have been reported to exacerbate cardiac effects of COVID-19 (Gao *et al.* 2023). JAK inhibitors have been explored for the treatment and management of COVID-19 (Levy *et al.* 2022) and is known to regulate the release of pro-inflammatory cytokines and chemokines. For instance, the chemokines *CXCL9, CXCL10* and *CXCL11* are known to be upregulated in the COVID-19 response (Callahan *et al.* 2021). IFIT1, IFIT2, and IFIT3 form a functional complex and participate in interferon-induced broad viral response (Mears and Sweeney 2018, Singh *et al.* 2022). *ISG15* encodes a ubiquitin-like protein whose activation triggers the release of various pro-inflammatory cytokines and chemokines (Cao 2021).

In addition to the enrichment of COVID-19-related genes that are consistent with literature, BoostDiff also identified edges between *TREX1* and *IFIT1, IFIT3, IFITM3, ISG15*, and *OAS3*, all of which have been implicated with COVID-19. While *TREX1* has not been directly linked to COVID-19 pathogenesis, it encodes an exonuclease that is involved in host defense and has also been implicated in autoimmune diseases (Stetson *et al.* 2008). In particular, *TREX1* regulates interferon-mediated signaling in antiviral response (Hasan *et al.* 2013). Interestingly, a recent genome-wide screen identified TREX1 as a pro-viral factor in viral replication during influenza infection (King *et al.* 2023). Given that nucleic acid detection is a key component of the immune response, *TREX1* could be a candidate player involved in immune response to viral pathogens, including SARS-CoV-2, that can be examined in future investigations.

#### 3.2.2 Crohn’s disease

**Figure 5.**
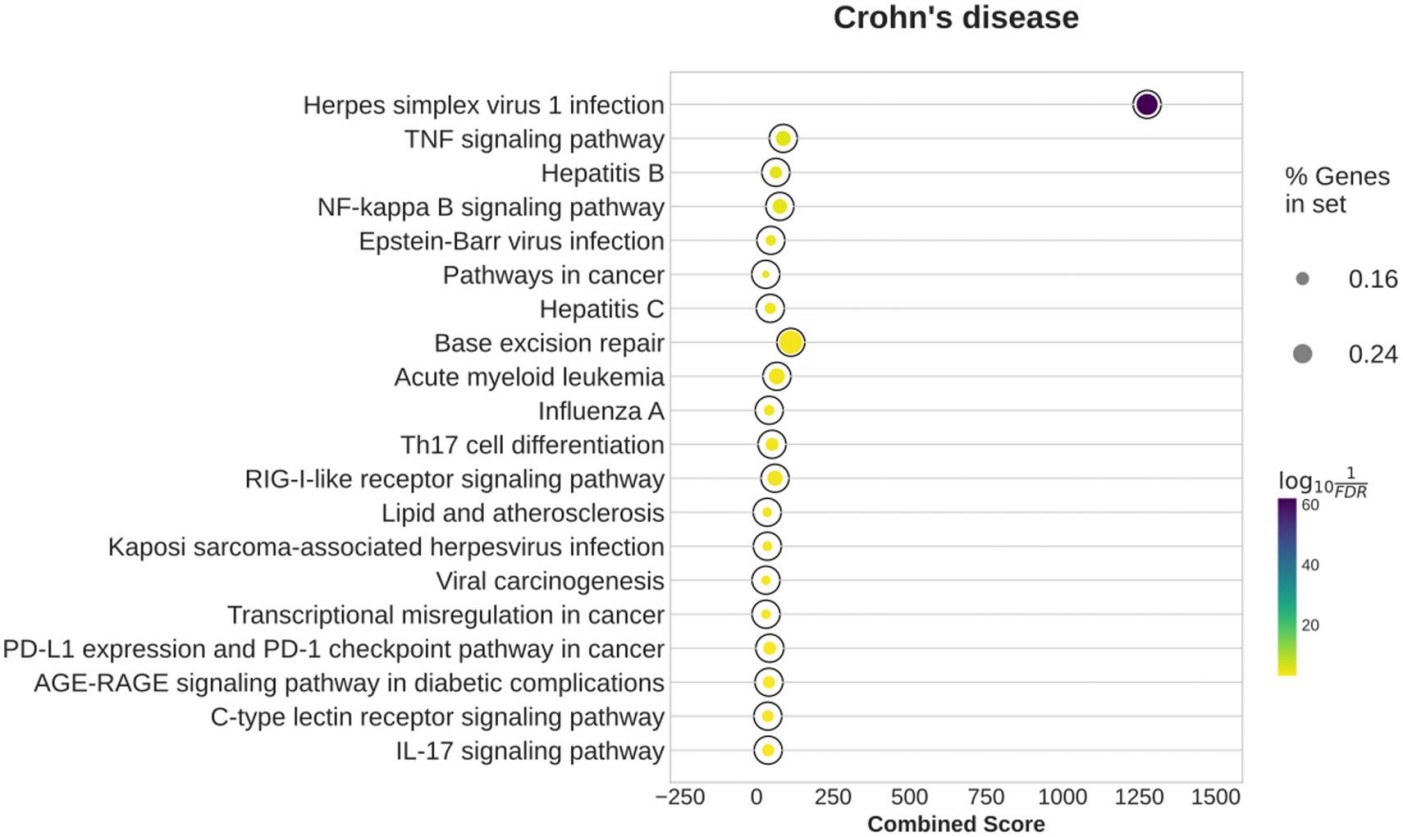
Enriched KEGG pathways in the networks inferred by BoostDiff for the CD dataset. The resulting differential networks are strongly enriched in inflammation-related and other relevant pathways, consistent with known disease pathophysiology.

Notably, the BoostDiff network shows stronger enrichment of CD-related pathways than the networks inferred by the other methods (Fig. 5 and Supplementary Tables S12–S15). The network returned by Differential GENIE3 does not show any significant enrichment. In addition to infection pathways, the network output by BoostDiff for the CD dataset is enriched in CD-relevant pathways, including ‘TNF signaling pathway,’ ‘NOD-like receptor signaling pathway,’ ‘Th17 cell differentiation,’ ‘IL-17 signaling pathway,’ and ‘NF-κB signaling,’ as well as ‘Inflammatory bowel disease.’ NOD-like receptors play important roles in host defense and homeostasis by acting as sensors of microbial pathogens in the intestinal mucosa. IBD has been associated with abnormal gut microbiota composition, which promotes NF-κB signaling and downstream inflammatory responses (Hug *et al.* 2018). The TF NF-κB functions in maintaining intestinal homeostasis, and dysregulation of the NF-κB pathway leads to sustained inflammatory state characteristic of IBD patients (Zaidi and Wine 2018). NF-κB signaling activation has been associated with more severe clinical manifestations in CD patients (Han *et al.* 2017, Zaidi and Wine 2018). The Th17 subset of ${\text{CD}4}^{+}$ T cells have well recognized roles in IBD pathogenesis. In CD, IL-17 signaling mediates the activation of Th17 cells, which further drive pro-inflammatory cascades via the production of IL-21, IL-22, IFN-γ, and TNF (Schmitt *et al.* 2021). The differential edges obtained from the two BoostDiff sub-analyses where the disease or control states are used as the target condition also show enrichment of CD-relevant pathways (Supplementary Fig. S13). The nodes in the BoostDiff network have a small overlap with the list of DEGs (Jaccard similarity=0.008). Enrichment results after removal of DEGs are similar to those of the original network (Supplementary Fig. S14). The Diffcoex network is enriched in only the term ‘Herpes simplex virus 1 infection,’ while the *z*-score network is enriched in many cancer-related terms (Supplementary Tables S12 and S13). The EBcoexpress network is also enriched in CD-related terms, such as ‘IL-17 signaling pathway,’ ‘NOD-like receptor signaling pathway,’ ‘TNF signaling pathway’ and ‘NF-κB signaling pathway.’ (Supplementary Table S15).

**Figure 6.**
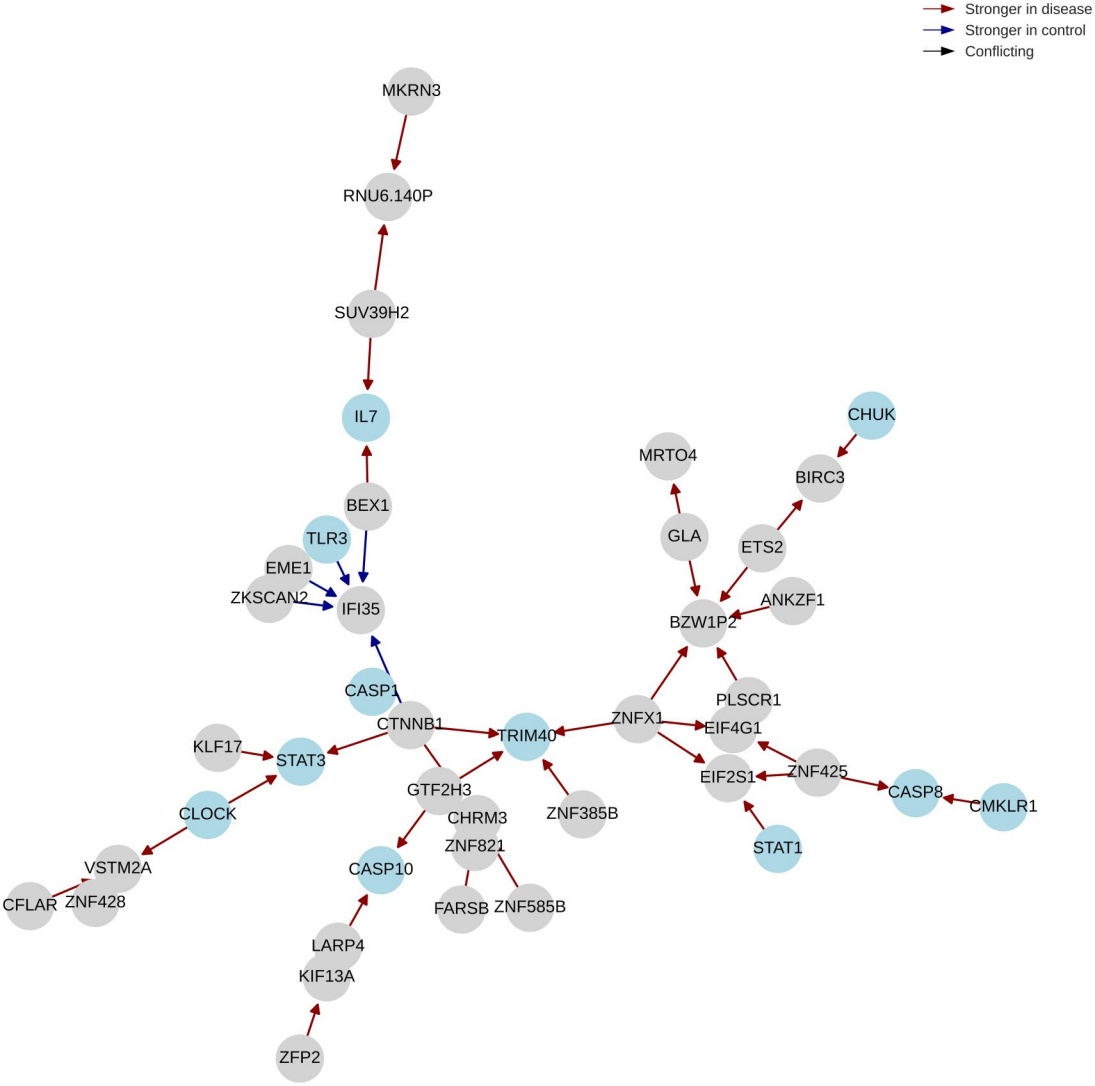
Dysregulated module identified from the CD differential network output by BoostDiff. Notable genes include *CLOCK, TRIM40, IL7, TLR3, CASP1, STAT3, STAT1, CASP8, CASP10, CMKLR1*, and *CHUK*.

The Louvain algorithm is then applied on the differential networks to refine the results. For Diffcoex and Differential GENIE3, none of the modules show significant CD-related enrichment. For *z*-score, one module is enriched in metabolism-related genes (Supplementary Fig. S15 and Supplementary Table S16). The EBcoexpress network contains a module that is enriched in inflammation-related terms ‘Natural killer cell mediated cytotoxicity,’ ‘NF-κB signaling pathway,’ and ‘TNF signaling pathway’ (Supplementary Fig. S16 and Supplementary Table S17). On the other hand, the BoostDiff network contains a module enriched in the terms ‘Necroptosis,’ ‘Apoptosis,’ and ‘TNF signaling pathway’ (Fig. 6 and Supplementary Table S18). CD is characterized by inflammation in the intestinal mucosa leading to necroptosis, autophagy, and apoptosis. TLRs, such as TLR3, are central players in microbial defense in the gut that trigger downstream inflammatory signaling pathways, such as NF-κB (Lu *et al.* 2018). The complex roles of caspases, including CASP1, CASP8, and CASP10, are also known to drive gut inflammation and tissue damage (Mandal *et al.* 2018). *CHUCK* is a member of the NF-κB signaling pathway that functions in chemokine signaling (Awane *et al.* 1999). Aberrant STAT1 and STAT3 signaling in IBD pathogenesis has been previously established (Nieminen *et al.* 2013). The protective role of chemokine-like receptor 1 (CMKLR1) against microbiota-driven colitis has been reported (Lin *et al.* 2022). *TRIM40* drives IBD pathogenesis by targeting cell-cell junctions in the mucosal epithelial barrier and promoting inflammation in the gut (Noguchi *et al.* 2011, Kang *et al.* 2023). Disturbances in the circadian rhythm are also linked to IBD manifestations (Giebfried and Lorentz 2023); for instance, *CLOCK* expression is disrupted in IBD patients (Weintraub *et al.* 2020, Giebfried and Lorentz 2023). Interestingly, BoostDiff identifies an edge between *CLOCK* and *STAT3*, which could point to a candidate regulatory mechanism where the circadian clock influences *STAT3*, a known driver of the CD phenotype. Thus, this dependency could point to further research in this direction.

#### 3.2.3 Stress response in *Bacillus subtilis*

BoostDiff also outperforms its competitors on the *B.subtilis* data (Supplementary Tables S19–S28). The networks inferred by BoostDiff are found to be enriched in stress-related pathways for all three conditions (Supplementary Fig. S17). On the other hand, only the Differential GENIE3 network in the salt stress condition is enriched in stress-related pathways. For Diffcoex, only one of the networks (heat treatment) is significantly enriched in stress-related pathways.

**Figure 7.**
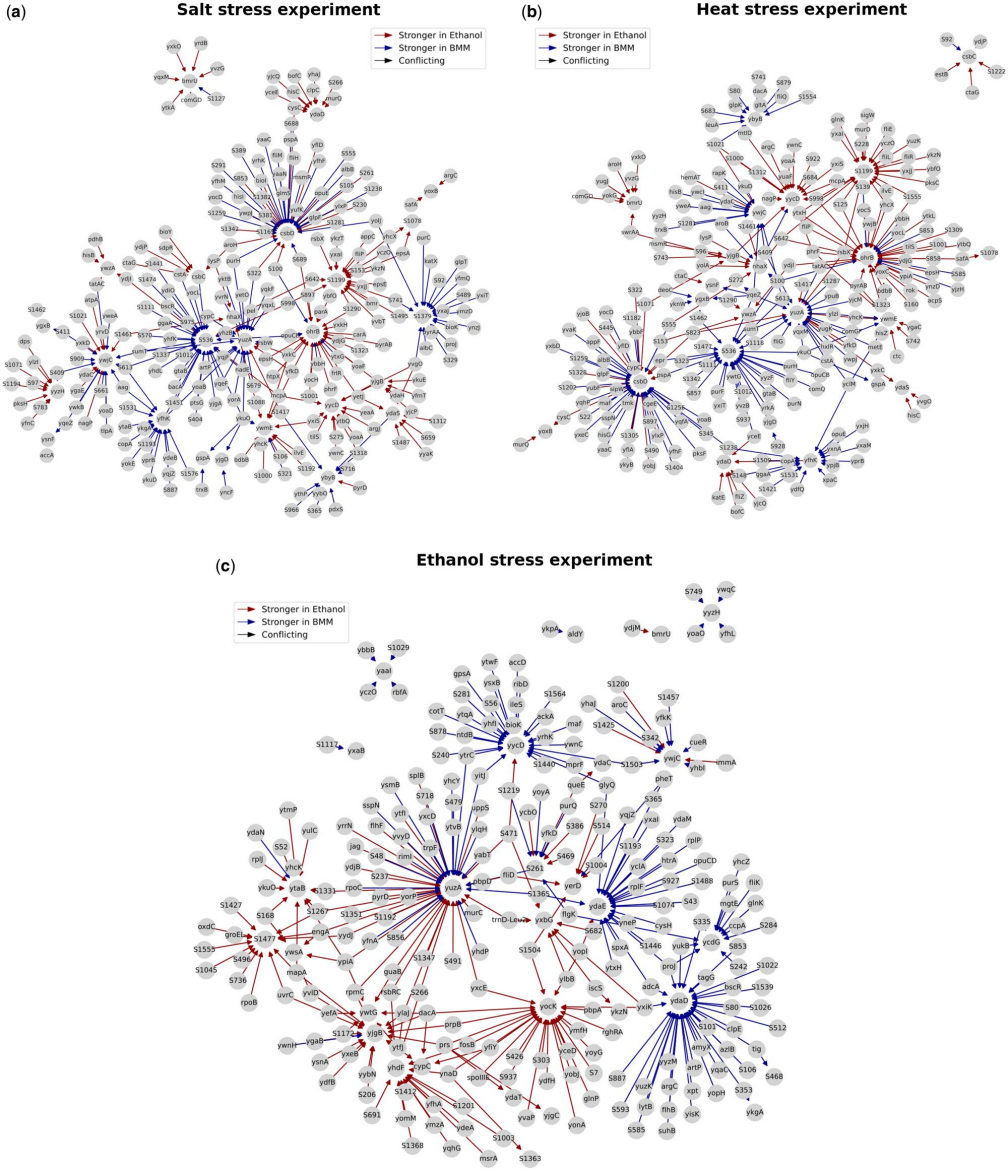
The differential networks identified by BoostDiff for the stress response experiments in *B.subtilis*. (a) The differential network inferred with the salt stress versus Spizizen’s minimal medium control comparison. (b) The differential network inferred with the heat stress versus Belitsky Minimal Medium (BMM) control comparison (c). The differential network inferred with the ethanol stress versus BMM control comparison. In all three conditions, the top ten hub genes identified by BoostDiff are members of the SigB regulon, which is induced in diverse stresses in *B.subtilis*.

Next, we examine the networks based on the top hub genes. For the salt stress experiment, all the top ten hub genes in the differential network (*csbD, S536, ohrB, yuzA, ywjC, S1199, S1379, yfhK, yycD, ywmE*, and *ybyB*) are part of the SigB regulon (Fig. 7). Similarly, for the heat stress experiment, BoostDiff identifies members of the SigB regulon, namely, *csbD, ohrB, S536, yuzA, S1199, yycD, ywjC, yfhK, ywmE*, and *nhaX*, as hub genes. For the ethanol stress experiment, BoostDiff idebtifies *yuzA, ydaD, yycD, ydaE, yocK, yjgB, yhdF, S1477*, and *ytaB* as the top ten hubs. Consistent with the applied stressors, the abovementioned genes encoding response proteins are part of the SigB regulon. The general stress response is controlled by the alternative sigma factor SigB, which induces the transcription of >100 genes across a wide range of stress stimuli (Nannapaneni *et al.* 2012). On the other hand, the other methods identify fewer relevant hub genes or do not produce meaningful outputs. EBcoexpress assigns a total of 68 532 edges and 1 080 620 edges with a posterior probability of 1.0 for the salt stress and heat stress experiments, respectively, thereby hindering biological interpretation. For the ethanol stress experiment, EBcoexpress did not run even after stringent filtering because of strong correlations in the dataset. The *z*-score method only identifies *ctc* as the relevant hub gene in the salt stress condition and no relevant genes in the heat stress condition; for the ethanol experiment, the *z*-score method identifies the ribosomal gene *rplJ* as a hub, which is not directly involved in the stress response (Supplementary Fig. S18). For Diffcoex, only three members of the SigB regulon (*ydbD, yqjL*, and *yvgO*) are identified as hubs in the heat stress condition and one relevant hub (*S1290*) is identified in the salt stress condition; no relevant hub gene is identified in the ethanol stress condition (Supplementary Fig. S19). Differential GENIE3 identifies only *yflD* as a hub in the salt stress condition (Supplementary Fig. S20).

#### 3.2.4 TCGA—breast cancer

**Figure 8.**
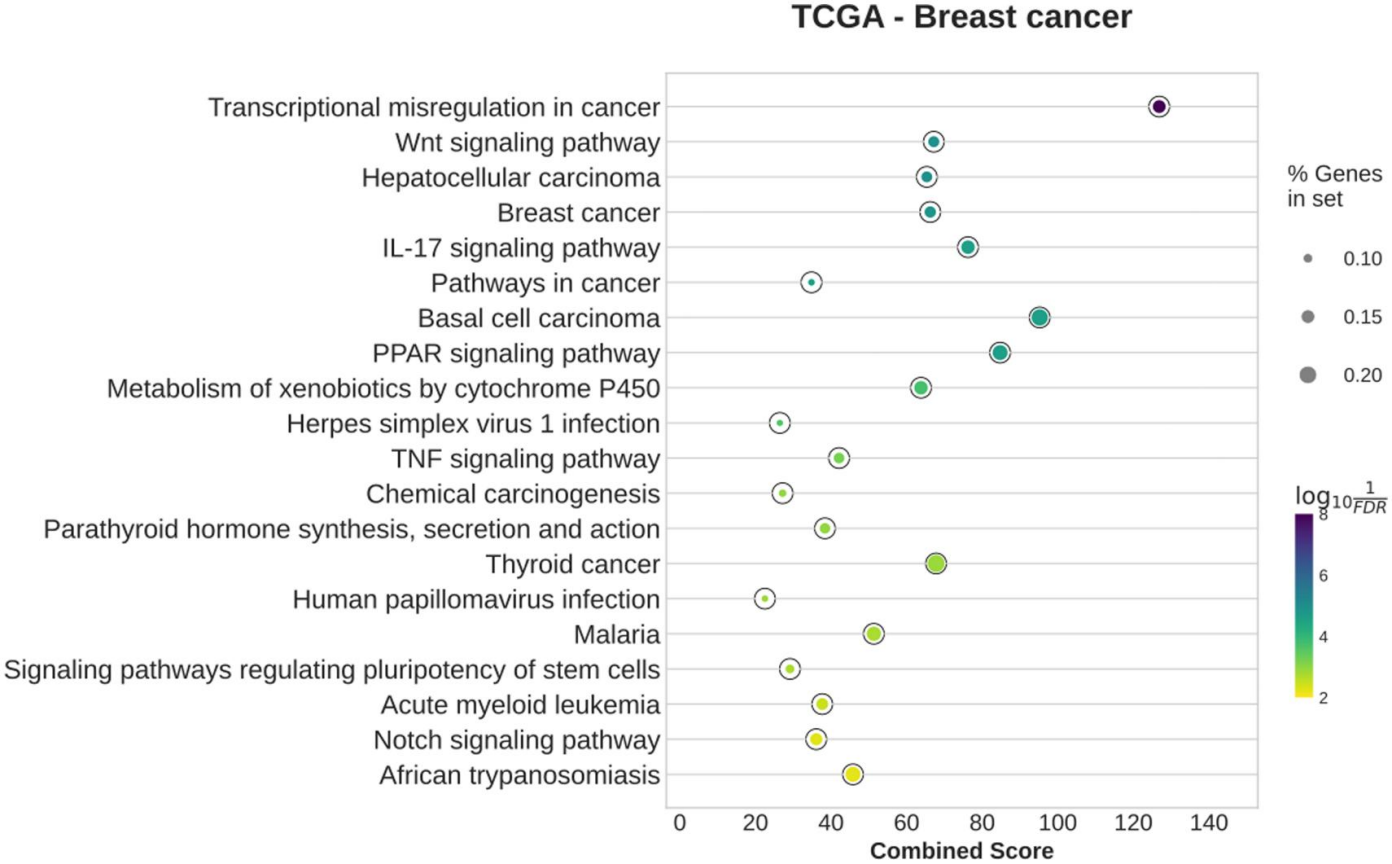
Enriched KEGG pathways in the networks inferred by BoostDiff for the TCGA BRCA dataset.

Again, the network inferred by BoostDiff shows stronger enrichment of disease-related pathways (including ‘Transcriptional misregulation in cancer,’ ‘Pathways in cancer,’ ‘Proteoglycans in cancer,’ and ‘Signaling pathways regulating pluripotency of stem cells’) than networks inferred using Diffcoex, EBcoexpress, Differential GENIE3, or the *z*-score method (Fig. 8 and Supplementary Tables S29–S32). Only the Diffcoex network is enriched in inflammatory and cancer-related pathways. Ebcoexpress assigns a posterior probability of 1 to 375 873 edges, while the *z*-score network is enriched in one term, ‘Tight junction’ (Supplementary Tables S30 and S31). The network inferred by Differential GENIE3 is enriched in terms related to olfactory transduction and metabolism (Supplementary Table S32). For BoostDiff, the results of enrichment analysis when considering the two sub-analyses separately shows generally similar results (Supplementary Fig. S21). The nodes in the BoostDiff network have a small overlap with the list of DEGs (Jaccard similarity=0.126). Enrichment results after removal of DEGs are similar to those of the original network (Supplementary Fig. S22).

**Figure 9.**
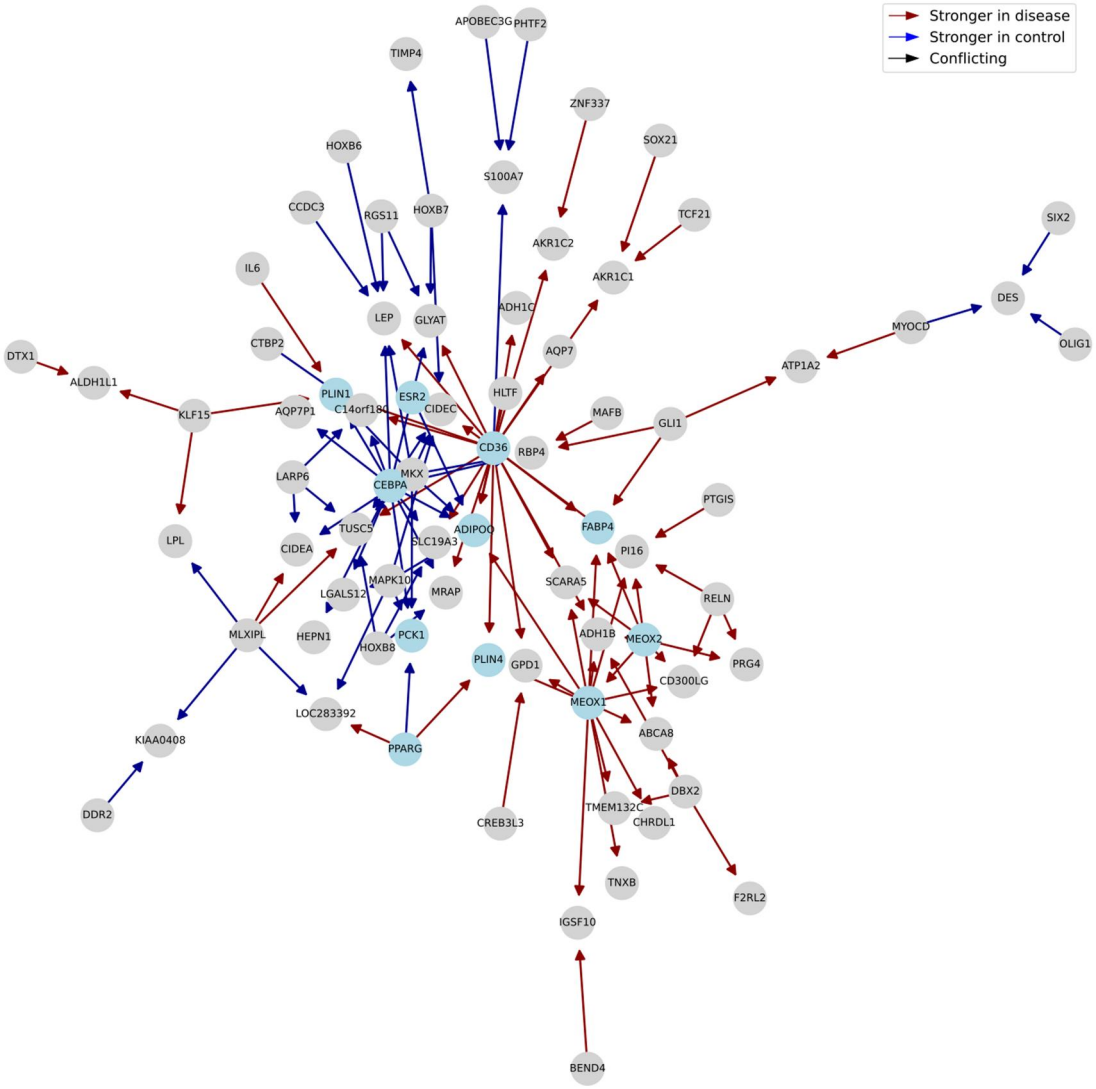
Dysregulated Louvain module for the TCGA BRCA dataset. Notable genes include *PPARG, PLIN1, PLIN4, CD36, ESR2, ADIPOQ, FABP4, PCK1, CEBPA, MEOX1*, and *MEOX2*.

No BRCA-related module is found in the Differential GENIE3 network. Module analysis for Diffcoex identifies a module that is primarily enriched in generic inflammation-related pathways, such as ‘IL-17 signaling pathway,’ ‘TNF signaling pathway,’ and ‘JAK-STAT signaling pathway.’ (Supplementary Fig. S23 and Supplementary Table S33). BoostDiff results are more specific: Here, we identify a module that is enriched in the terms ‘PPAR signaling pathway,’ ‘Insulin resistance,’ ‘AMPK signaling pathway,’ ‘Adipocytokine signaling pathway,’ ‘Pyruvate metabolism,’ and ‘Regulation of lipolysis in adipocytes’ (Fig. 9 and Supplementary Table S34). Adipose tissue plays an important role in tumor progression because it provides nutrients, metabolites, and adipokines to the tumor microenvironment of breast cancer cells (DeBerardinis *et al.* 2008, Guaita-Esteruelas *et al.* 2017). PPAR is a ligand-activated TF that is involved in fatty acid oxidation and lipid metabolism (Lee *et al.* 2019). In the context of BRCA, PPAR signaling is known to contribute to metastasis and promote survival in harsh metabolic conditions (Saez *et al.* 2004, Wang *et al.* 2016).

The BoostDiff results not only make sense in light of the enriched pathways, but also when zooming-in on the individual edges and hub nodes: For instance, BoostDiff identifies a relationship between *PPARG* and *PLIN4*, which are already known to be associated in the context of adipogenesis (Nielsen *et al.* 2008). Perilipins comprise lipid droplet-associated proteins that modulate lipid homeostasis. In BRCA, *PLIN4* upregulation is associated with poor prognosis (Kim *et al.* 2015, Zhang *et al.* 2021). BoostDiff also predicts an edge between *PPARG* and *PCK1*, which is also part of the PPAR pathway. *PCK1* encodes a protein kinase that has multiple known metabolic roles, including lipogenesis and tumorigenesis (Tang *et al.* 2021). BoostDiff identifies *CD36* as a hub gene in the module. In BRCA, *CD36* has been reported to be involved in metabolic rewiring and reprogramming favoring fatty acid oxidation and has been proposed as a therapeutic target in breast cancer (Feng *et al.* 2019). BoostDiff further identified an edge between *CD36* and *FABP4*. In BRCA, CD36 was shown to directly interact with FABP4 to influence transport and metabolism of fatty acids (Gyamfi *et al.* 2021). Further, *CD36* is upregulated along with other lipid metabolism genes *PLIN1* and *ADIPOQ* (adiponectin) in breast cancer (Marino *et al.* 2020).

The correlation between *ESR2* and *ADIPOQ* in adipose tissue is also established (Ahmed *et al.* 2022). While this specific relationship has not been described in breast cancer, this could be investigated in future studies, given the established role of estrogens and adiposity in breast cancer (Bhardwaj and Brown 2021). Along with *PPARG, CEBPA* is a known master regulator of adipogenesis that influences lipid accumulation (Lee *et al.* 2019). Here, *CEBPA* is associated with *ADIPOQ*, another adipocyte marker (Matulewicz *et al.* 2017). Other identified TFs include *MEOX1* and *MEOX2*, which are involved in angiogenesis and vascular development (Schönrock *et al.* 2022, Wang *et al.* 2022, Zeng *et al.* 2023). For instance, *MEOX1* expression was found to be associated with tumor growth triple-negative BRCA (Zeng *et al.* 2023). Based on their emerging roles in neoplastic diseases, MEOX1 and MEOX2 could be candidate targets in breast cancer. Overall, our results identify genes that can be further explored and reinforce the important role of adipogenesis in breast cancer.

#### 3.2.5 TCGA—prostate adenocarcinoma

**Figure 10.**
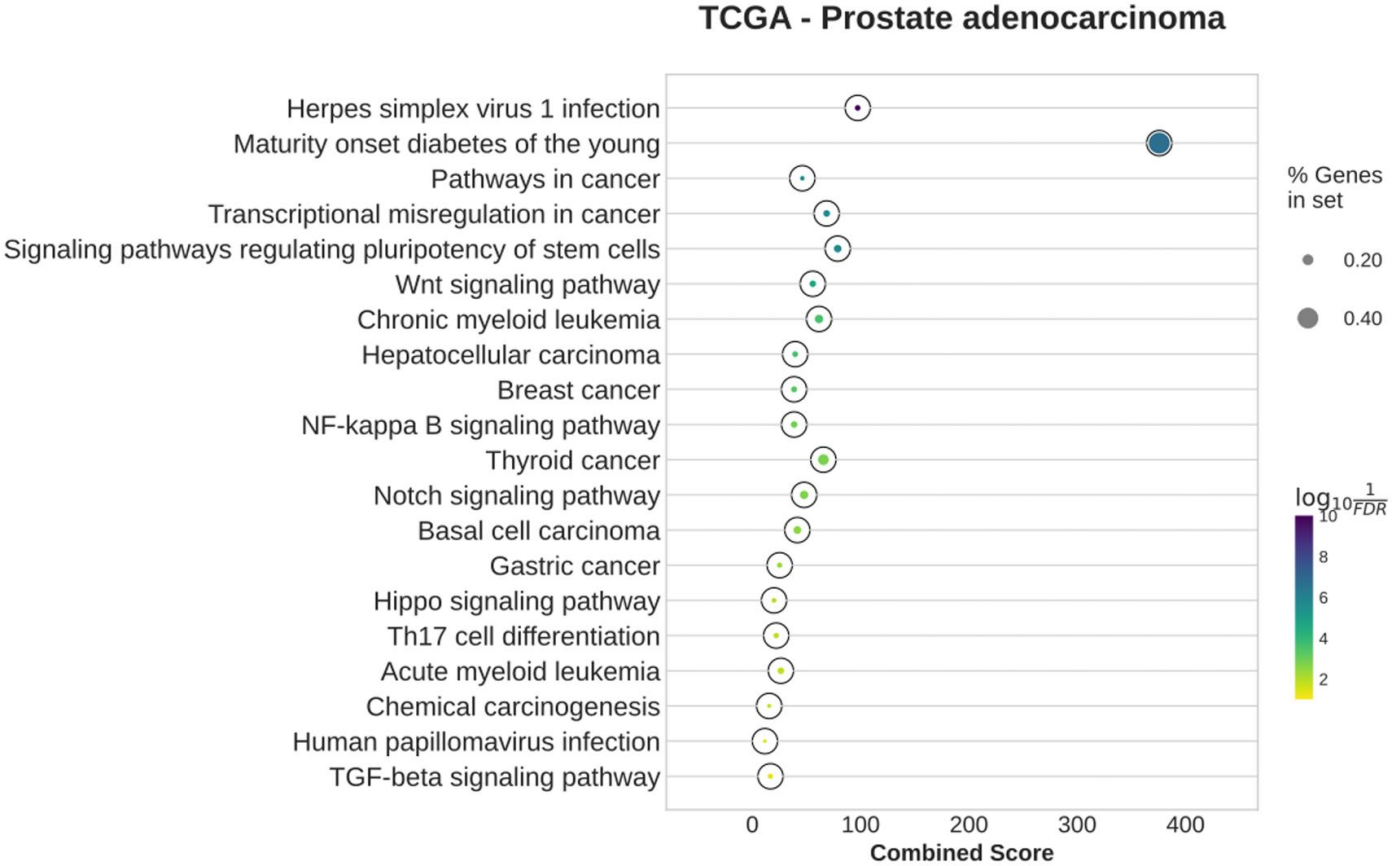
Enriched KEGG pathways in the networks inferred by BoostDiff for the TCGA PRAD dataset.

For PRAD, the EBcoexpress network is too large to analyze, as 375 873 edges are assigned with posterior probability of 1, while the *z*-score network is not significantly enriched in any terms. The Diffcoex network is enriched in infection- and cancer-related terms, while the Differential GENIE3 network is enriched in terms related to metabolism. On the other hand, the BoostDiff network shows overall stronger enrichment of cancer-related terms (Fig. 10 and Supplementary Tables S35–S37). The nodes in the BoostDiff network have a small overlap with the list of DEGs (Jaccard similarity=0.114). The results of enrichment analysis when considering the two sub-analyses separately shows generally similar results (Supplementary Fig. S24). Enrichment results after removal of DEGs are similar to those of the original network (Supplementary Fig. S25).

**Figure 11.**
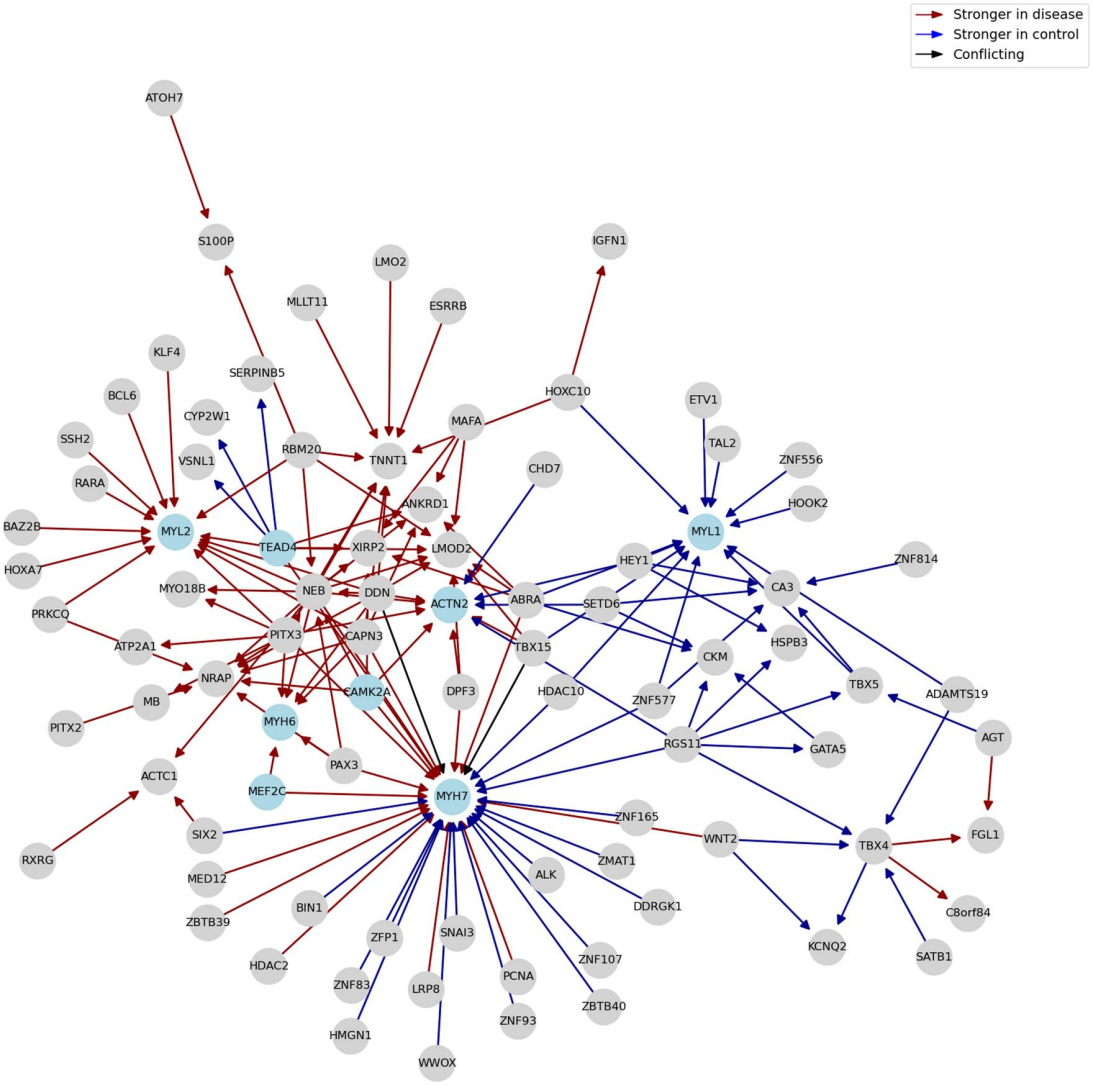
Dysregulated Louvain module for the TCGA PRAD dataset. Notable genes include *MEF2C, MYL1, MYH6, MYH7, MYL2, ACTN2, CAMK2A*, and *TEAD4*.

For Diffcoex, we identify a cancer-associated module enriched in terms such as ‘Cell cycle,’ ‘Cellular senescence,’ and ‘Bladder cancer’ (Supplementary Fig. S26 and Supplementary Table S38). None of the modules in the *z*-score and Differential GENIE3 networks show significant enrichment of cancer-related terms. For BoostDiff, we identify a module enriched in the terms ‘Transcriptional misregulation in cancer,’ ‘Adrenergic signaling in cardiomyocytes,’ ‘Dilated cardiomyopathy,’ ‘Thyroid hormone signaling pathway,’ ‘Cardiac muscle contraction,’ and ‘Pathways in cancer’ (Fig. 11 and Supplementary Table S39). The module contains MEF2C, a TF known to be involved in oncogenesis and tumor proliferation in many types of cancers (Bai *et al.* 2015, Di Giorgio *et al.* 2017). *TEAD4* was found to be an important prognostic marker in PRAD and is a known effector of oncogenic pathways (Chen *et al.* 2021). The potential involvement of *MYH7* in PRAD has been recently described; in particular (Liang *et al.* 2022), found a higher frequency of *MYH7* variants in prostate cancer patients. *CAMK2A* is known to promote tumor progression in prostate cancer (Rokhlin *et al.* 2007, He and Li 2021). In an earlier study, protein-protein interaction network-based analyses similarly identified *ACTN2, MYL1, MYL2*, and *MYH7* as hub genes associated with *PTEN*-associated prostate cancer (Sun *et al.* 2019). The dysregulation of *ACTN2* and *MYH7* has been described in the context of other cancers (Ju *et al.* 2023). Our results suggest that candidate genes in the identified module can be further examined in the context of prostate cancer.

#### 3.2.6 Expression distributions of top-ranked edges identified by BoostDiff reflect shifts in correlation distributions

**Figure 12.**
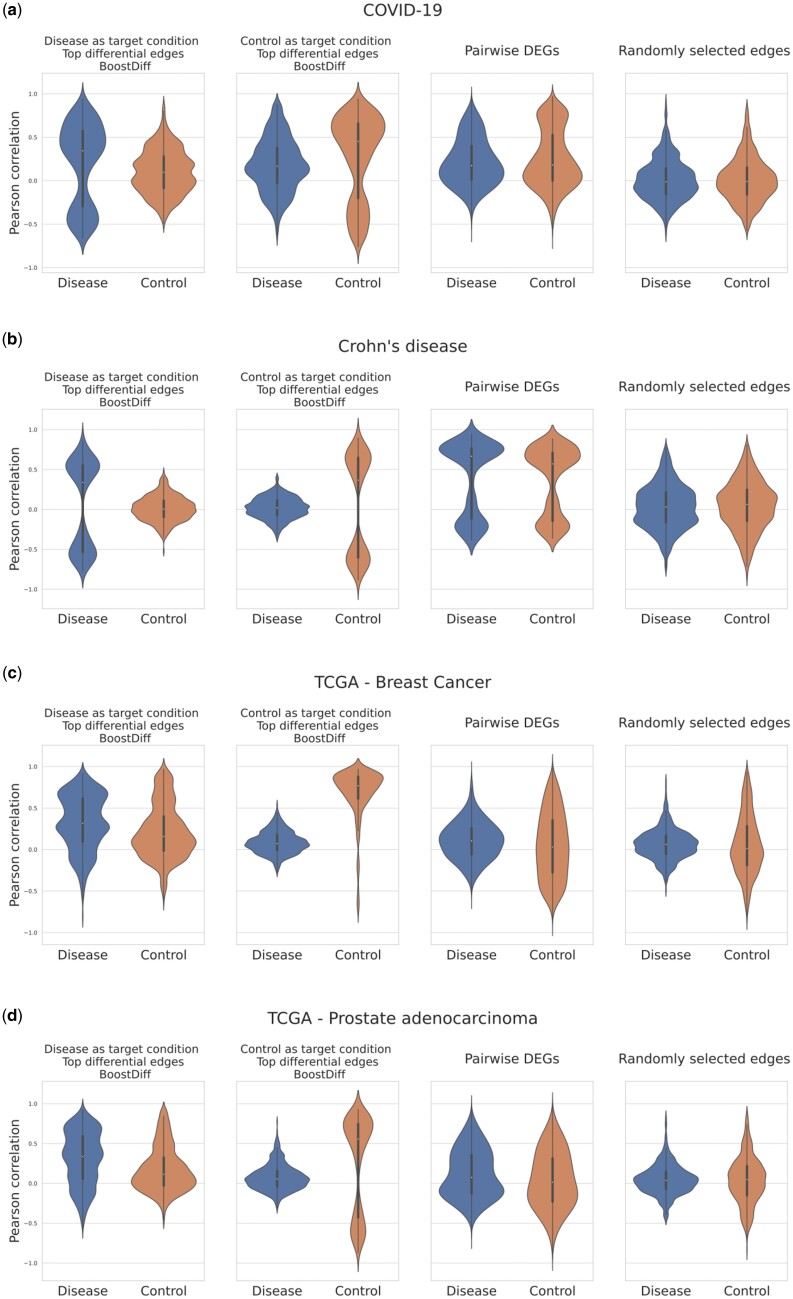
Violin plots showing that the top 500 edges in the differential network predicted by BoostDiff for each sub-analysis tend to exhibit changes in correlation distributions between the disease and control expression data, indicating dysregulation in pairwise relationships. Correlations between predicted differential edges are compared to correlations between all pairwise combinations of DEGs, as well as randomly selected edges. Results are shown for (a) COVID-19, (b) CD, (c) TCGA—BRCA, and (d) TCGA—PRAD datasets.

We additionally examine the Pearson correlations of the top edges from the differential networks identified by BoostDiff using the original expression data. This procedure is performed separately for the results of the two sub-analyses, namely, when the disease condition is used as the target condition, and when the control condition is used as the target condition. For the COVID-19 and CD datasets, for the same set of edges, we observe a unimodal distribution of correlation values in the nontarget condition and a bimodal distribution in the target condition, where strong positive correlation values suggest target-condition-specific activating regulator-target relationship, while negative values indicate target-condition-specific inhibitory relationships (Fig. 12). Similar observations can be derived for the *B.subtilis* stress adaptation and TCGA datasets (Supplementary Fig. S27). That is, expression levels of genes incident with differential edges returned by BoostDiff tend to exhibit highly condition-specific correlation patterns, indicating that BoostDiff can indeed uncover disease-specific gene dysregulation. Importantly, this striking observation does not hold for the set of all pairwise edges whose endpoints are DEGs or for randomly selected edges. For Differential GENIE3, similar patterns were observed for the salt stress and ethanol stress networks (Supplementary Fig. S28). However, no consistent patterns are observed when the same analysis is performed on the networks inferred from the human datasets (Supplementary Fig. S29).

## 4 Conclusions

Gene regulation is a complex process that changes under different biological contexts. Differential network biology explores the rewiring of these regulatory interaction landscapes that are fundamentally distinct from the static networks that are inferred in most standard GRN inference methods (Ideker and Krogan 2012). By additionally considering the regulatory dependencies from a baseline condition, we can uncover a more refined picture underlying the molecular processes that are perturbed in a condition of interest, such as disease.

Inference of networks from biological expression data is a challenging task. The novelty of BoostDiff is 2-fold: (i) We use differential variance improvement as the splitting measure in a tree-based algorithm that can explicitly compare two datasets with a continuous output variable; (ii) BoostDiff adapts the AdaBoost algorithm to use differential trees as the base learner. Boosting the differential trees with respect to samples belonging to the target condition is a crucial step that significantly improves the detection of differential edges.

BoostDiff outperforms existing differential network methods on simulated data in more complex settings with higher dimensionality. BoostDiff yields biologically meaningful results when evaluated on five real-world transcriptomics datasets. We show that BoostDiff infers differential networks that are consistent with the pathophysiology of COVID-19, Crohn’s disease, and two types of cancer, namely, breast cancer and prostate adenocarcinoma, using TCGA data. We further validate BoostDiff using three different stress conditions evaluating the response of *B.subtilis* to salt, heat, and ethanol stressors, where BoostDiff correctly identifies members of the SigB regulon as hub genes in the differential networks. On the other hand, the compared methods recover few stress-related response genes. BoostDiff is also scalable and has reasonable runtime since it builds one model for each gene and can hence easily be parallelized. To facilitate biological interpretation and further refine the results, various graph-based algorithms can be used to find highly connected subnetworks. In this study, we apply the Louvain community detection algorithm, although other graph-based tools can also be used to find perturbed modules.

Nevertheless, our method has several limitations. First, BoostDiff can only compare two conditions at a time. Moreover, BoostDiff is similar to GENIE3 in that it does not perform statistical testing. Instead, scores are assigned to individual edges by calculating tree-based variable importance measures; thus, only the ranking of the edge weights is considered. Further, the AdaBoost algorithm can be prone to overfitting. In our experiments, BoostDiff performance decreased when setting the number of trees to 300. Thus, to avoid overfitting, we recommend users to set a low number of base differential trees (e.g. 50 trees).

The application of BoostDiff is not limited to gene expression data; the proposed feature selection approach can be generalized to other omics datasets. For instance, BoostDiff can be applied to proteomics or metabolomics studies that aim to detect changes in dependencies of proteins or metabolites. Moreover, the simple but effective strategy implemented in BoostDiff is an algorithmic advancement that can be further extended to other problems that aim to extract differentially predictive features. Adapting BoostDiff for analyzing time-series datasets is also a promising research direction.

## Supplementary Material

vbae034_Supplementary_Data

## Data Availability

The data used in this article are publicly available. Transcriptomics data used for analyzing the COVID-19, Crohn’s disease, and *Bacillus subtilis* stress response can be downloaded from Gene Expression Omnibus under the accession numbers GSE156063, GSE126124, and GSE27219, respecively. The TCGA datasets are available at https://xenabrowser.net/datapages/. The simulated gene expression data are deposited at https://zenodo.org/records/10214950. Our BoostDiff implementation, as well as sample scripts and tutorials are available on GitHub: https://github.com/scibiome/boostdiff_inference.
