# Supplementary figures and images for: Inference of differential gene regulatory networks using boosted differential trees
Source: Bioinform Adv. 2024 Feb 29;4(1):vbae034. doi: 10.1093/bioadv/vbae034 (PMC10948285; doi:10.1093/bioadv/vbae034)

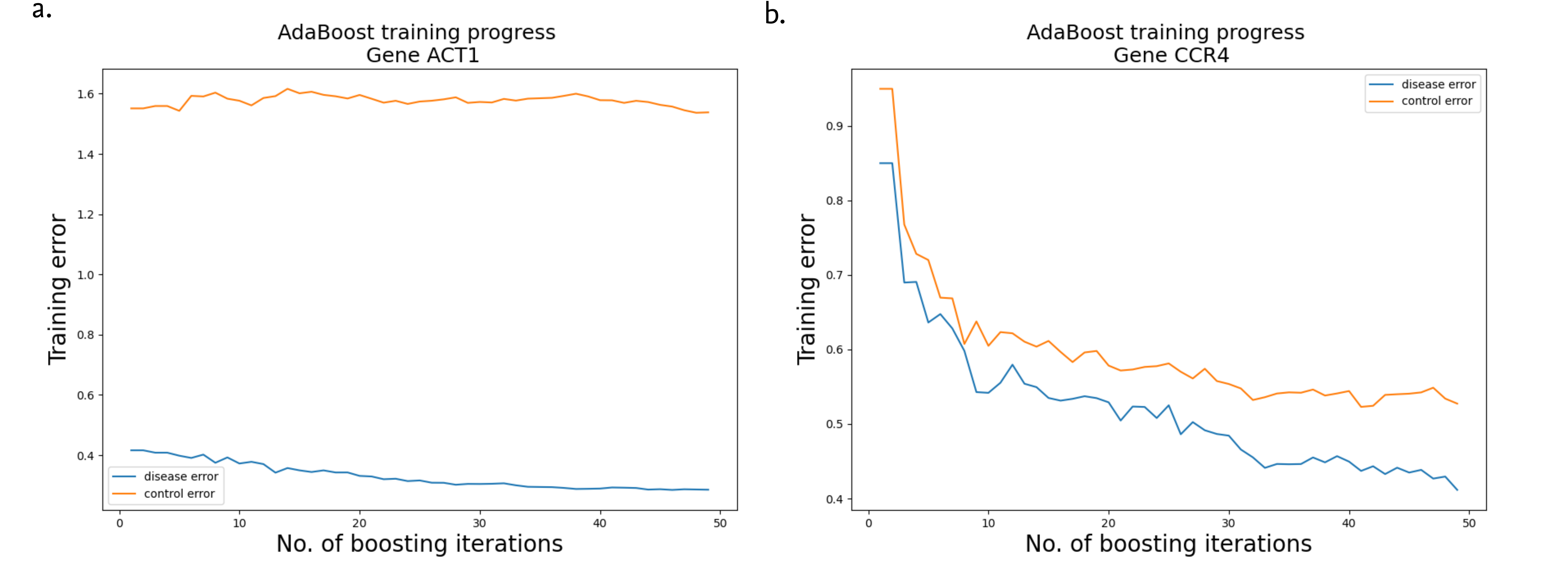

Supplement: vbae034_Supplementary_Data [file vbae034_supplementary_data.zip › FigS1_adaboost_training_progress.png]

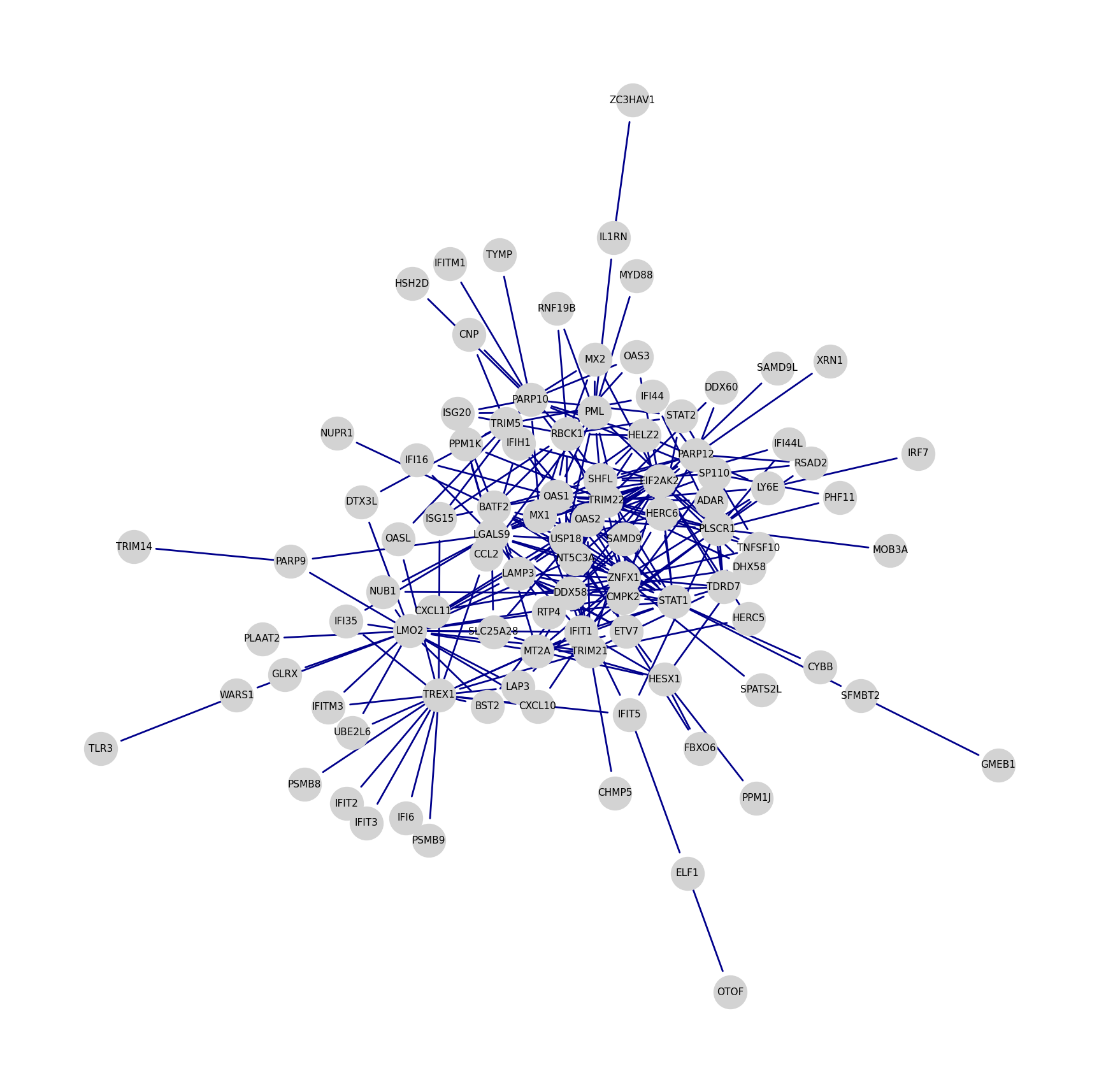

Supplement: vbae034_Supplementary_Data [file vbae034_supplementary_data.zip › FigS10_covid19_diffcoex_module.png]

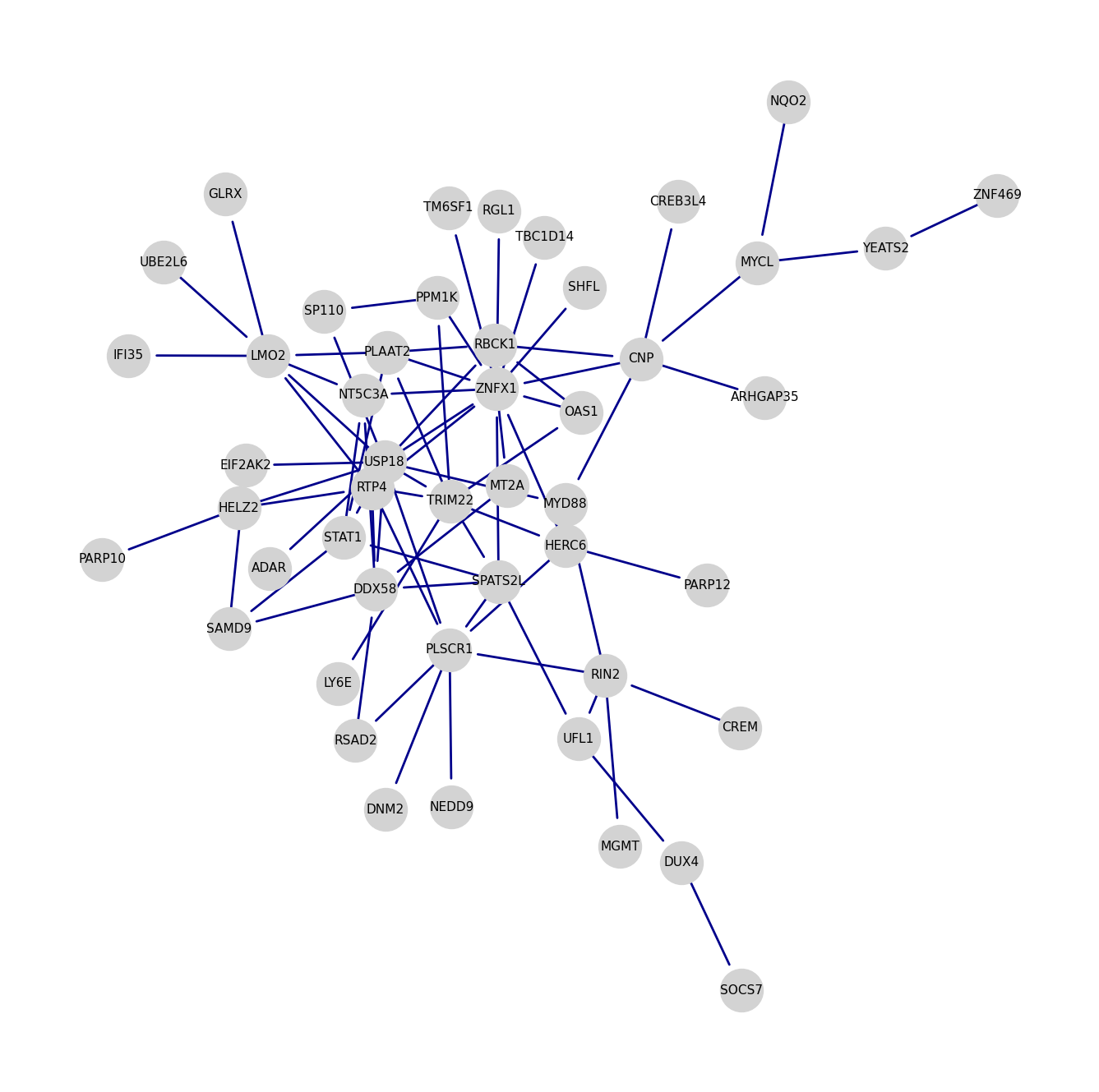

Supplement: vbae034_Supplementary_Data [file vbae034_supplementary_data.zip › FigS11_covid19_zscore_module.png]

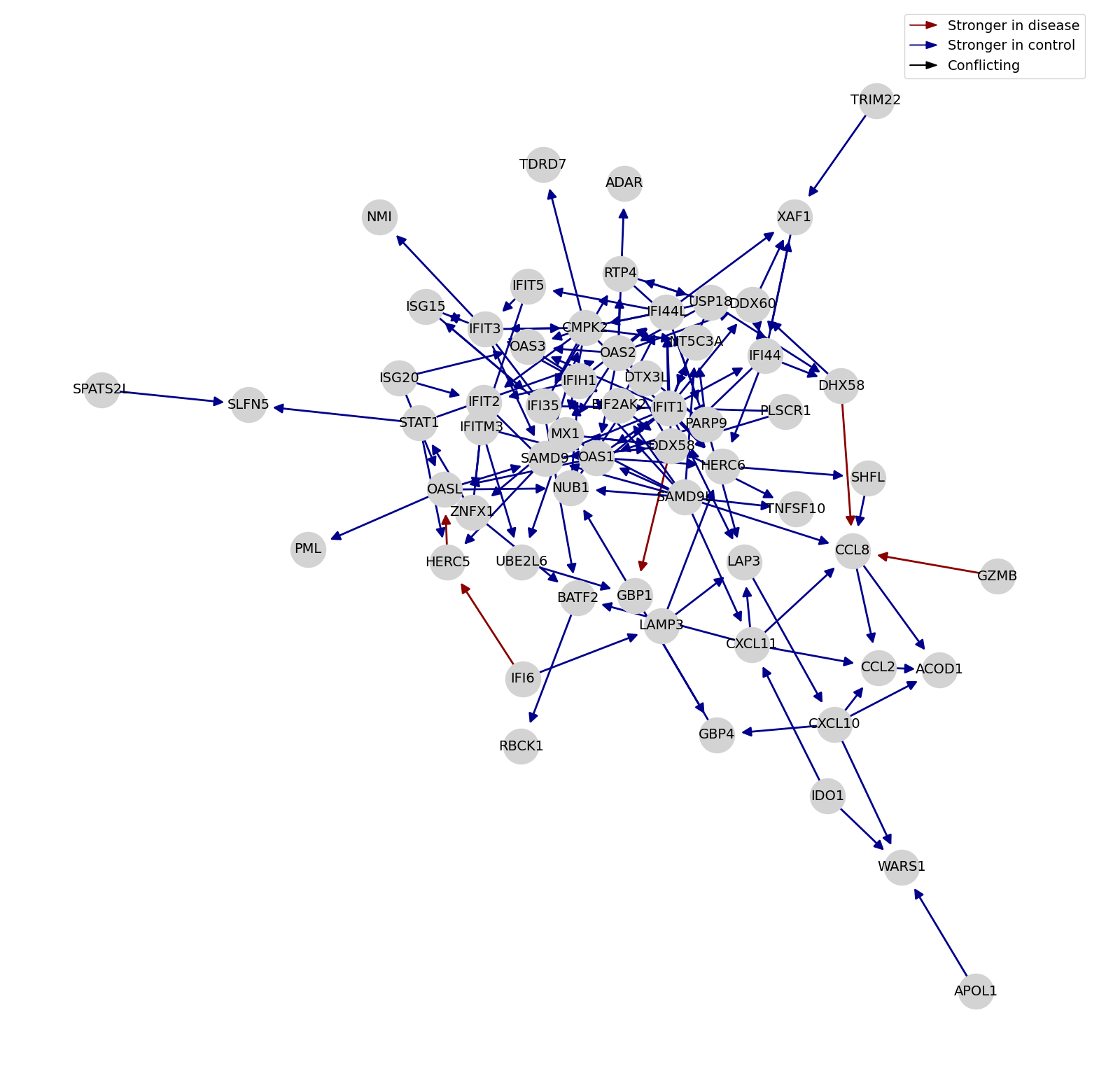

Supplement: vbae034_Supplementary_Data [file vbae034_supplementary_data.zip › FigS12_covid19_diffgenie3_module.png]

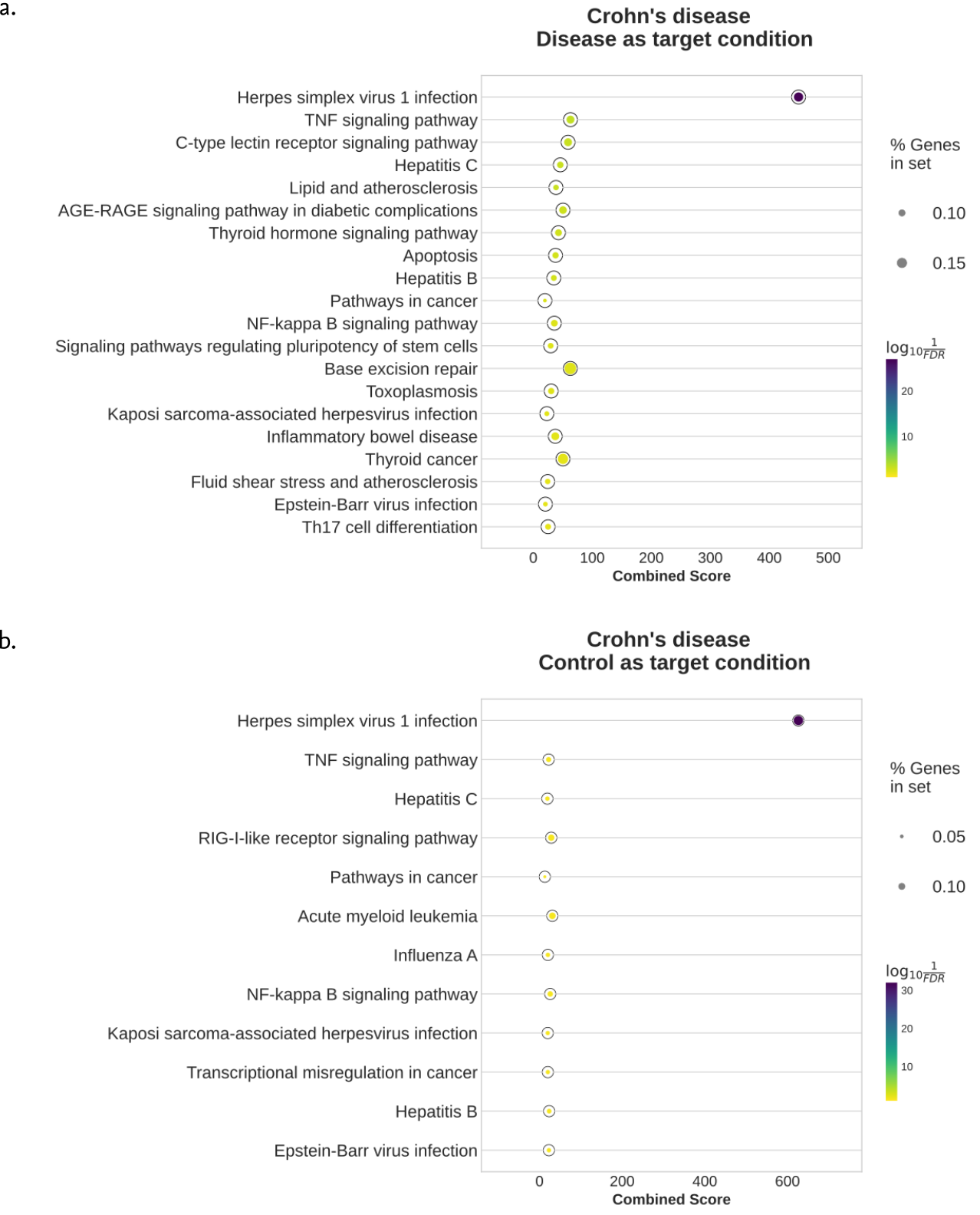

Supplement: vbae034_Supplementary_Data [file vbae034_supplementary_data.zip › FigS13_crohns_disease_target_conditions.png]

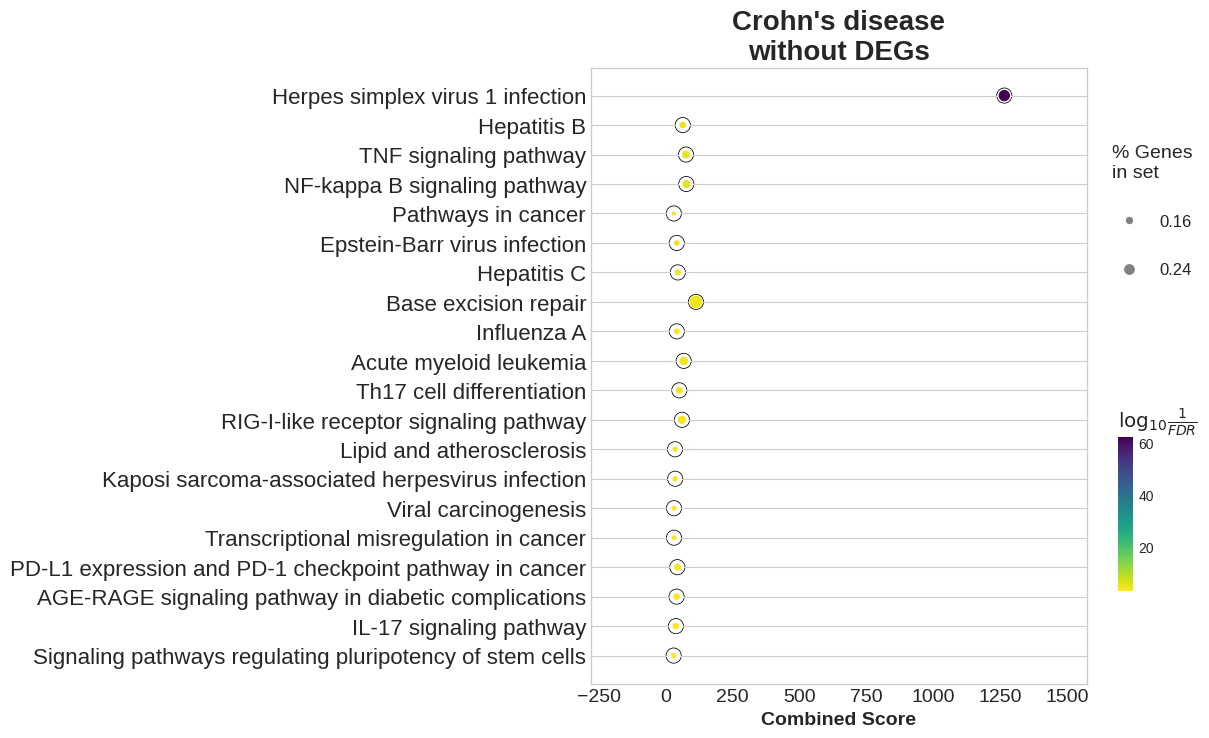

Supplement: vbae034_Supplementary_Data [file vbae034_supplementary_data.zip › FigS14_crohns_without_degs.png]

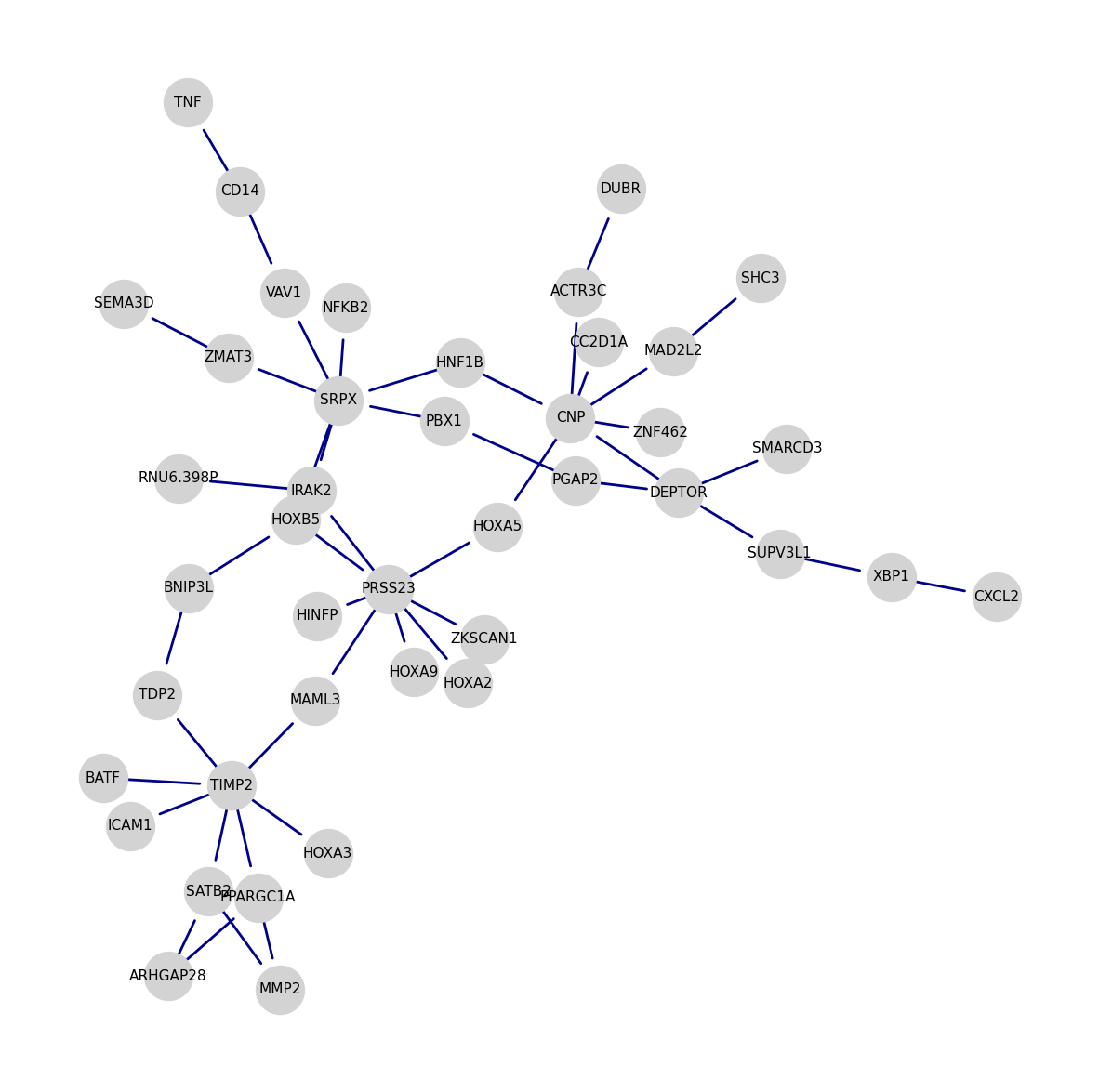

Supplement: vbae034_Supplementary_Data [file vbae034_supplementary_data.zip › FigS15_crohns_ebcoexpress_module.png]

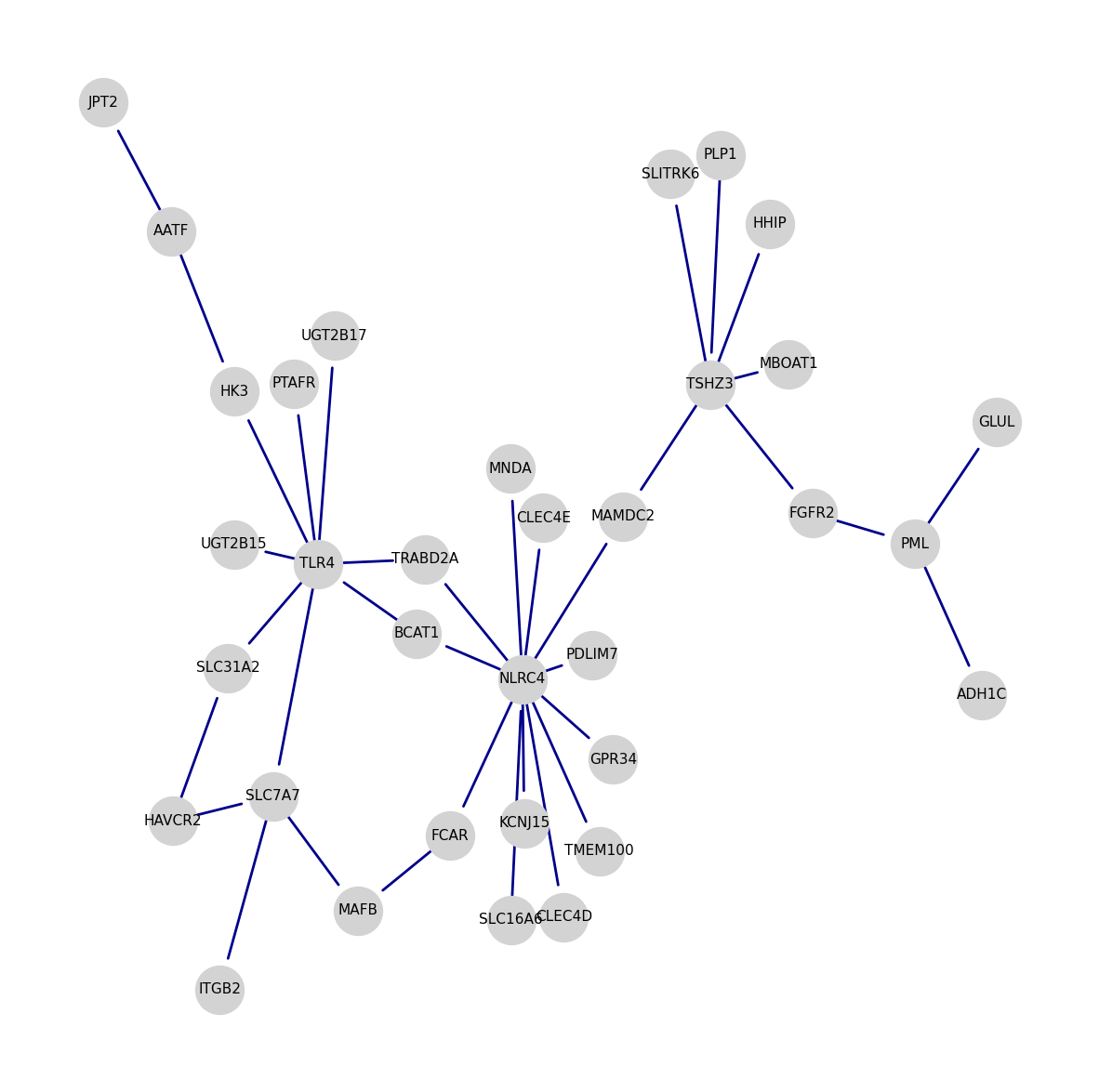

Supplement: vbae034_Supplementary_Data [file vbae034_supplementary_data.zip › FigS16_crohns_zscore_module.png]

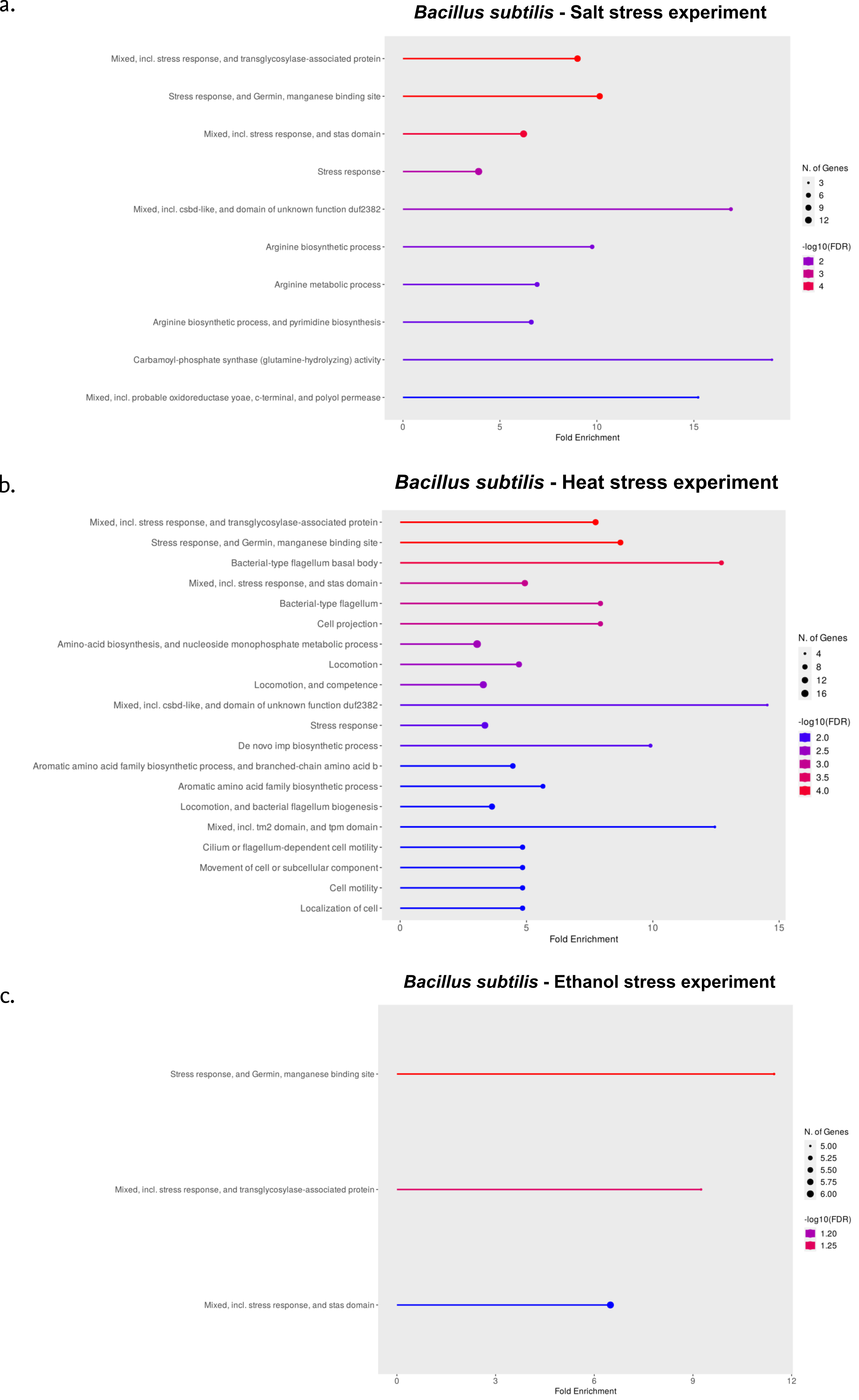

Supplement: vbae034_Supplementary_Data [file vbae034_supplementary_data.zip › FigS17_bsubt_enrichment.png]

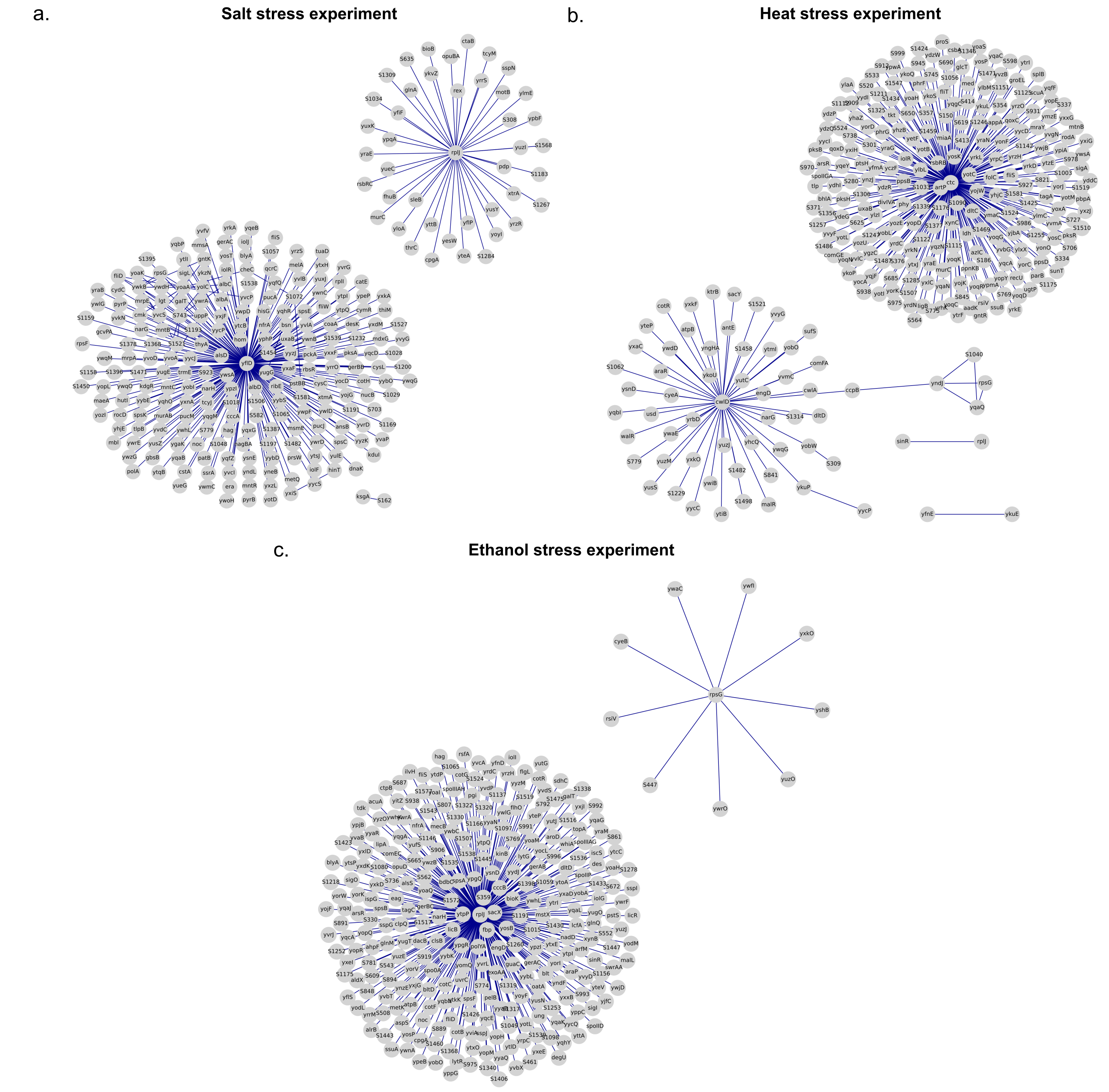

Supplement: vbae034_Supplementary_Data [file vbae034_supplementary_data.zip › FigS18_b_subtilis_zscore_salt_heat_stress.png]

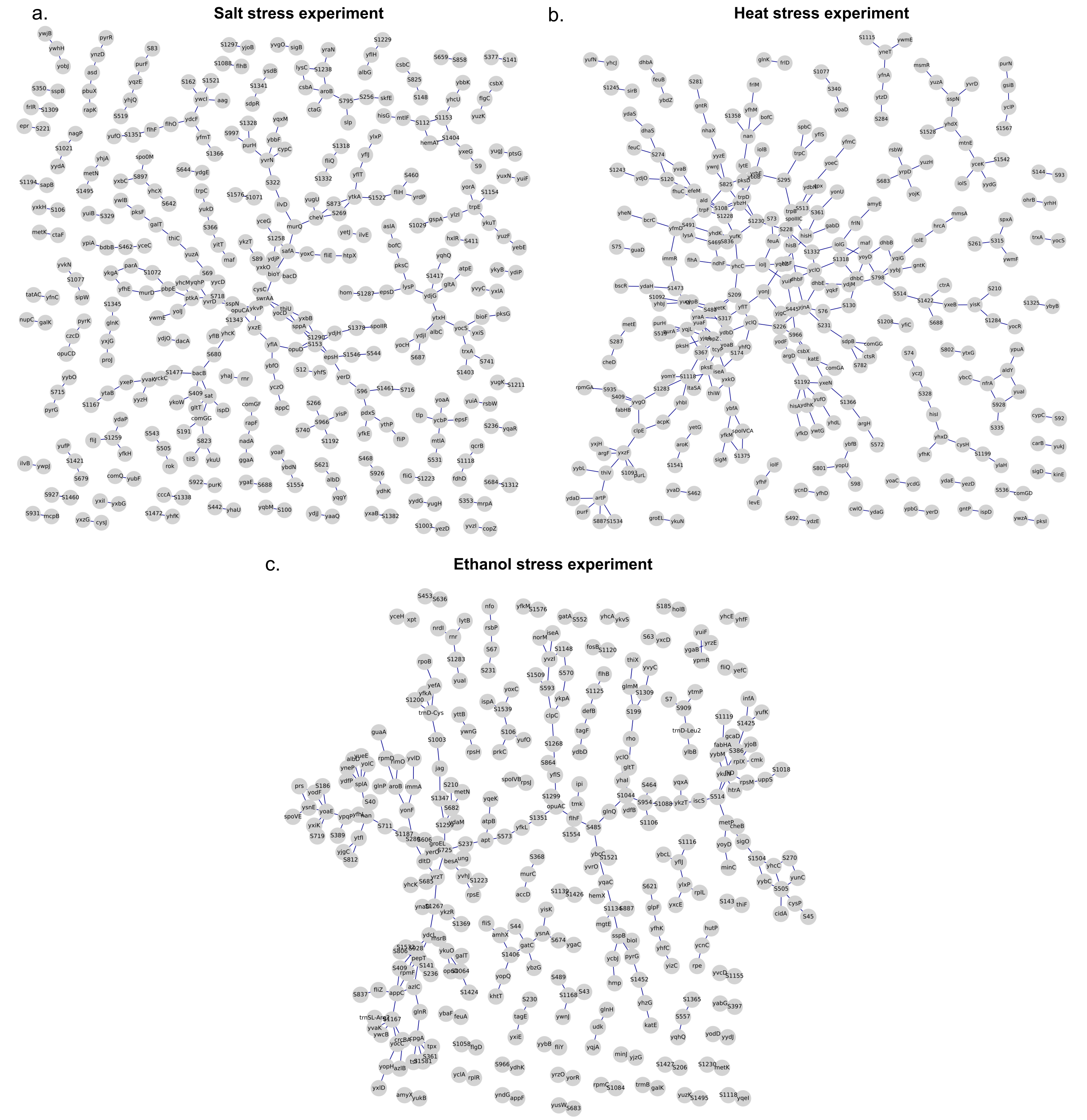

Supplement: vbae034_Supplementary_Data [file vbae034_supplementary_data.zip › FigS19_diffcoex_salt_heat_ethanol_stress.png]

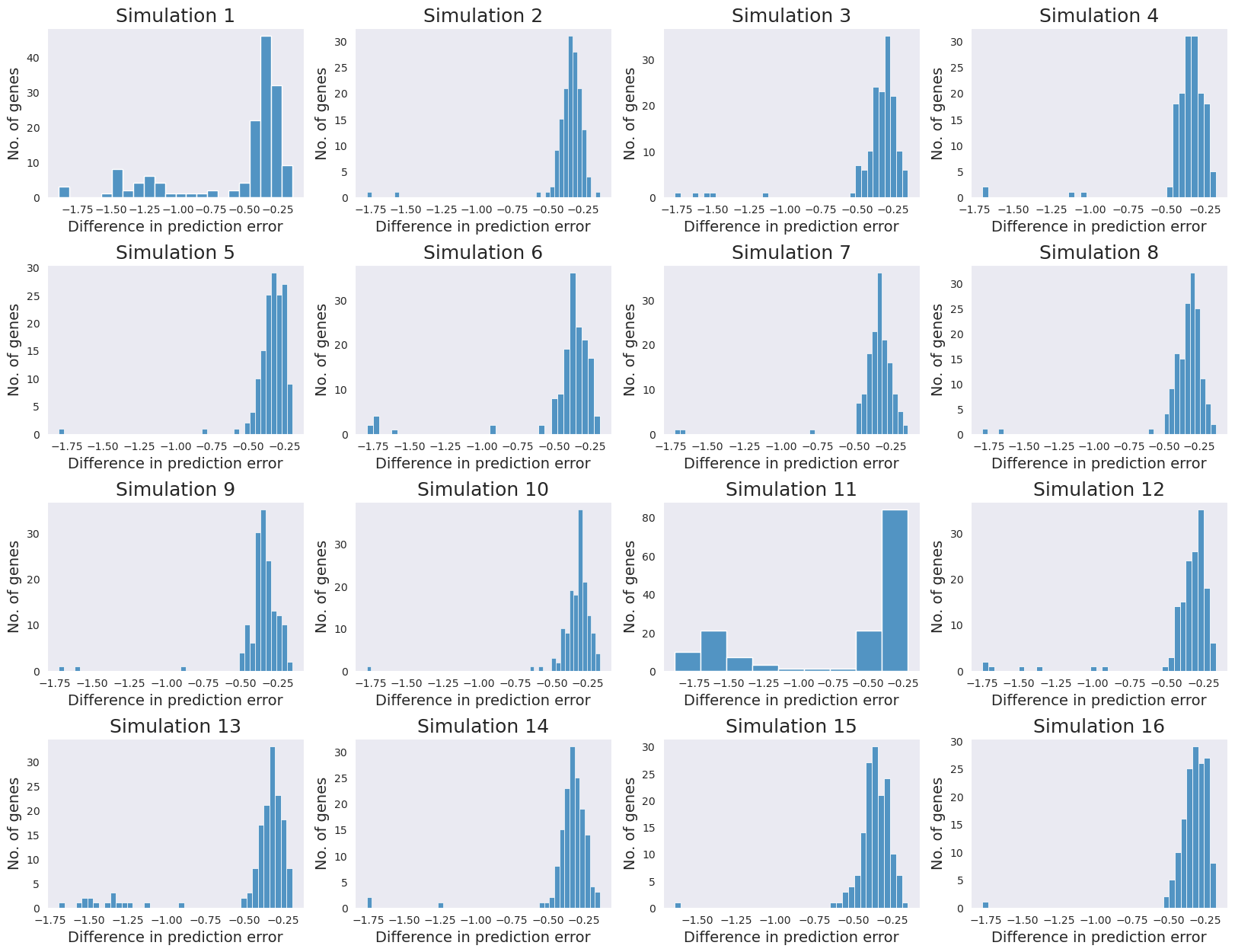

Supplement: vbae034_Supplementary_Data [file vbae034_supplementary_data.zip › FigS2_histograms_diff_training_error_simulated.png]

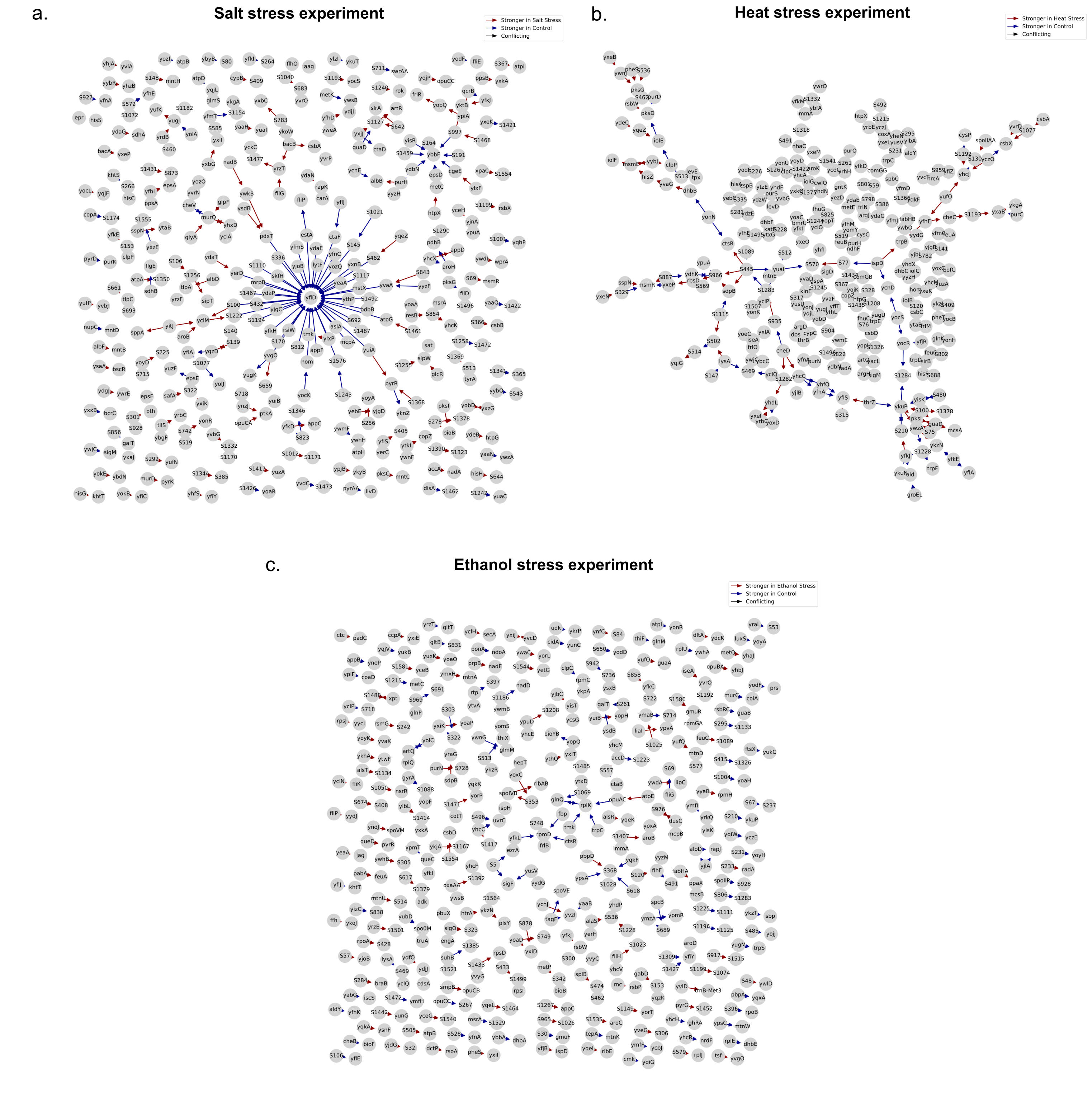

Supplement: vbae034_Supplementary_Data [file vbae034_supplementary_data.zip › FigS20_diffgenie3_salt_heat_ethanol_stress.png]

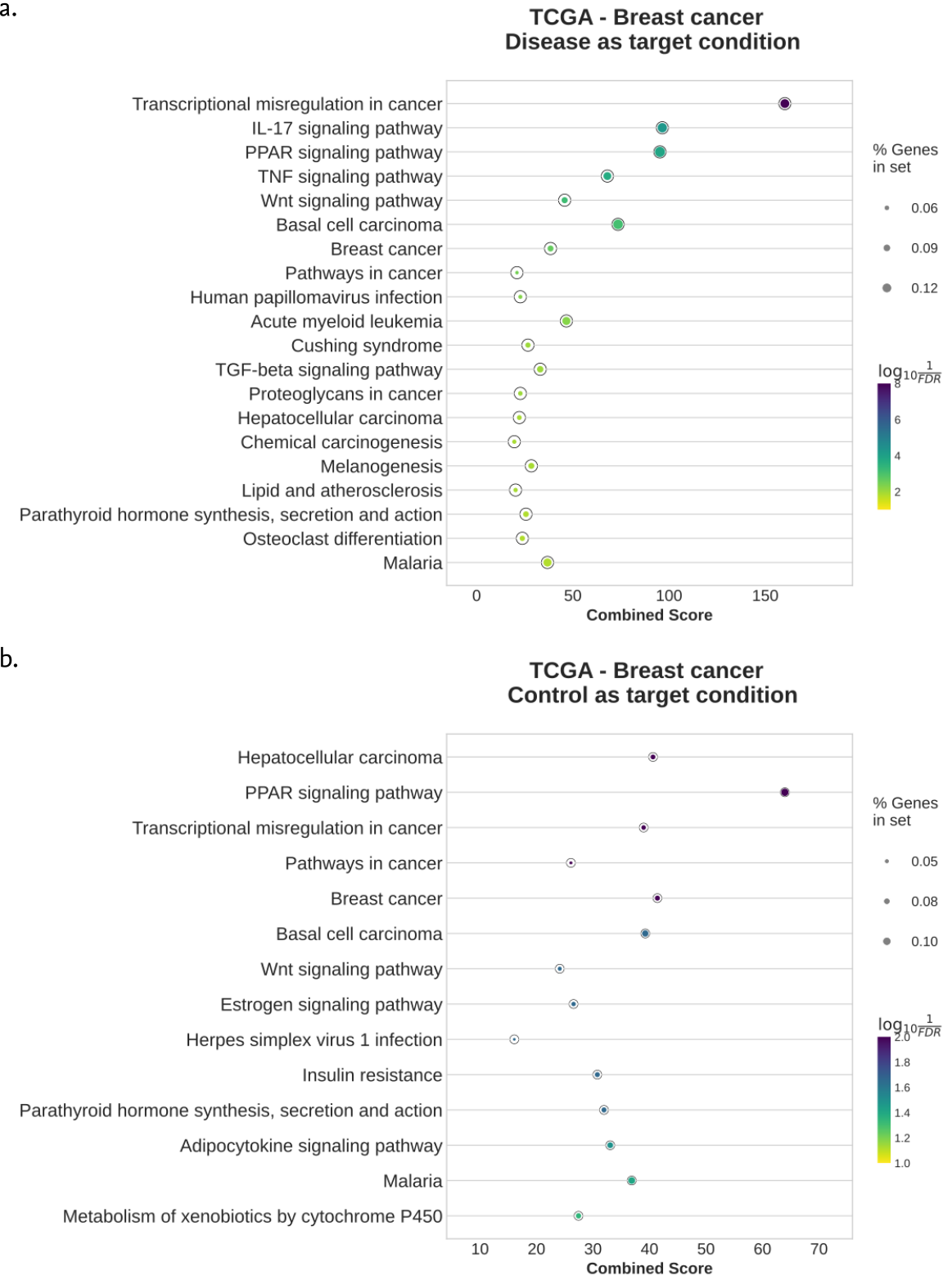

Supplement: vbae034_Supplementary_Data [file vbae034_supplementary_data.zip › FigS21_brca_target_conditions.png]

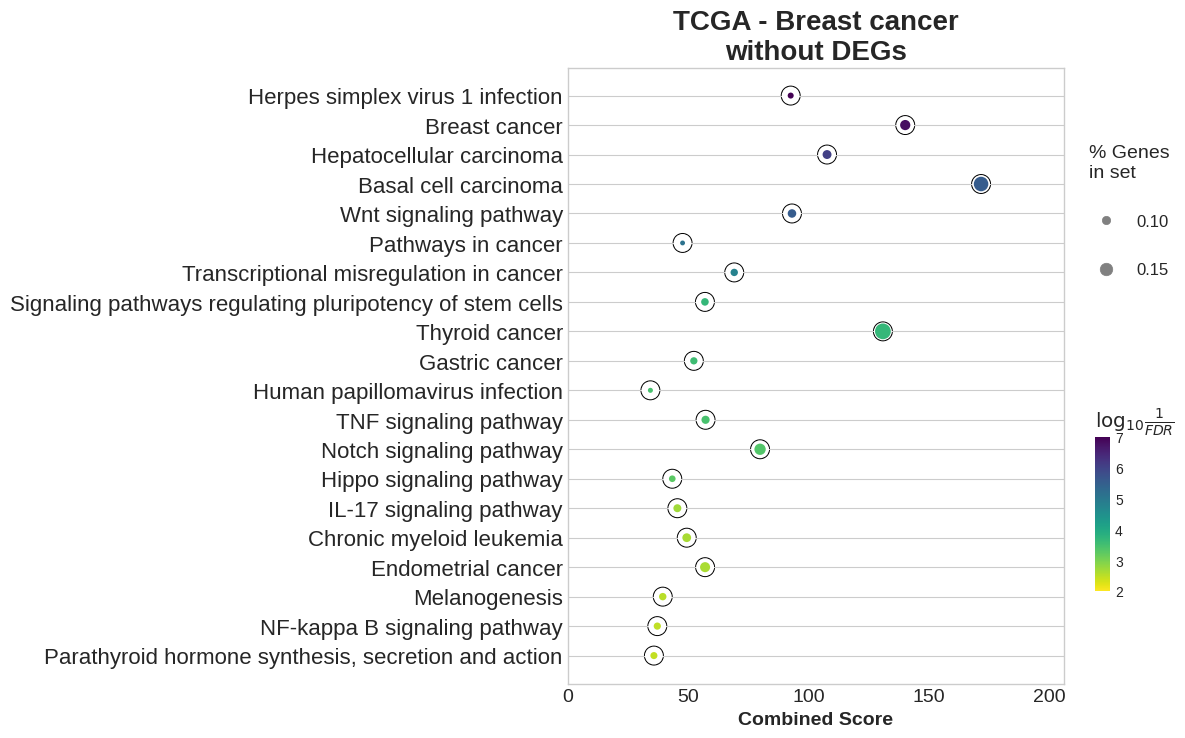

Supplement: vbae034_Supplementary_Data [file vbae034_supplementary_data.zip › FigS22_TCGA_BRCA_without_degs.png]

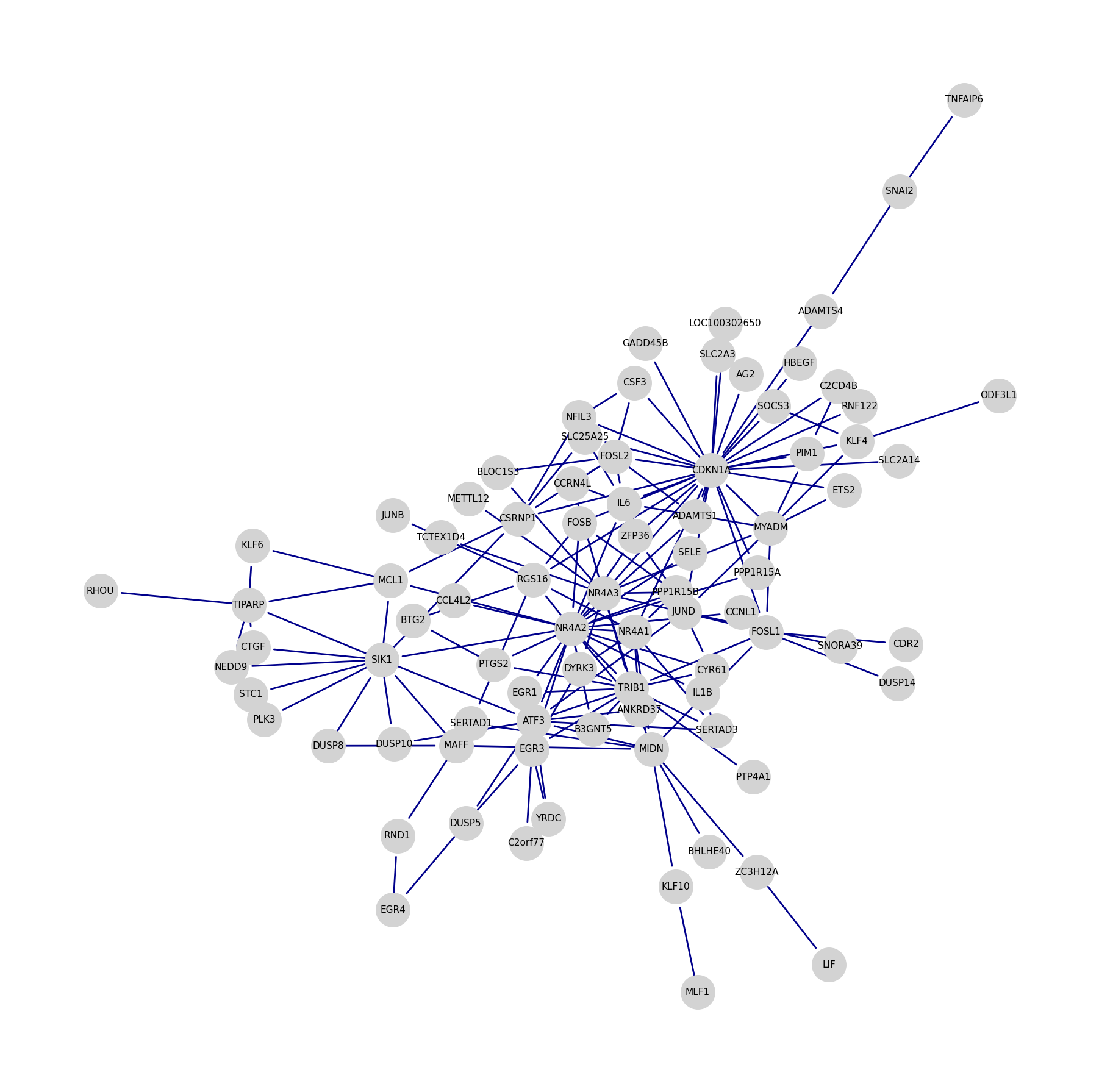

Supplement: vbae034_Supplementary_Data [file vbae034_supplementary_data.zip › FigS23_brca_diffcoex_module.png]

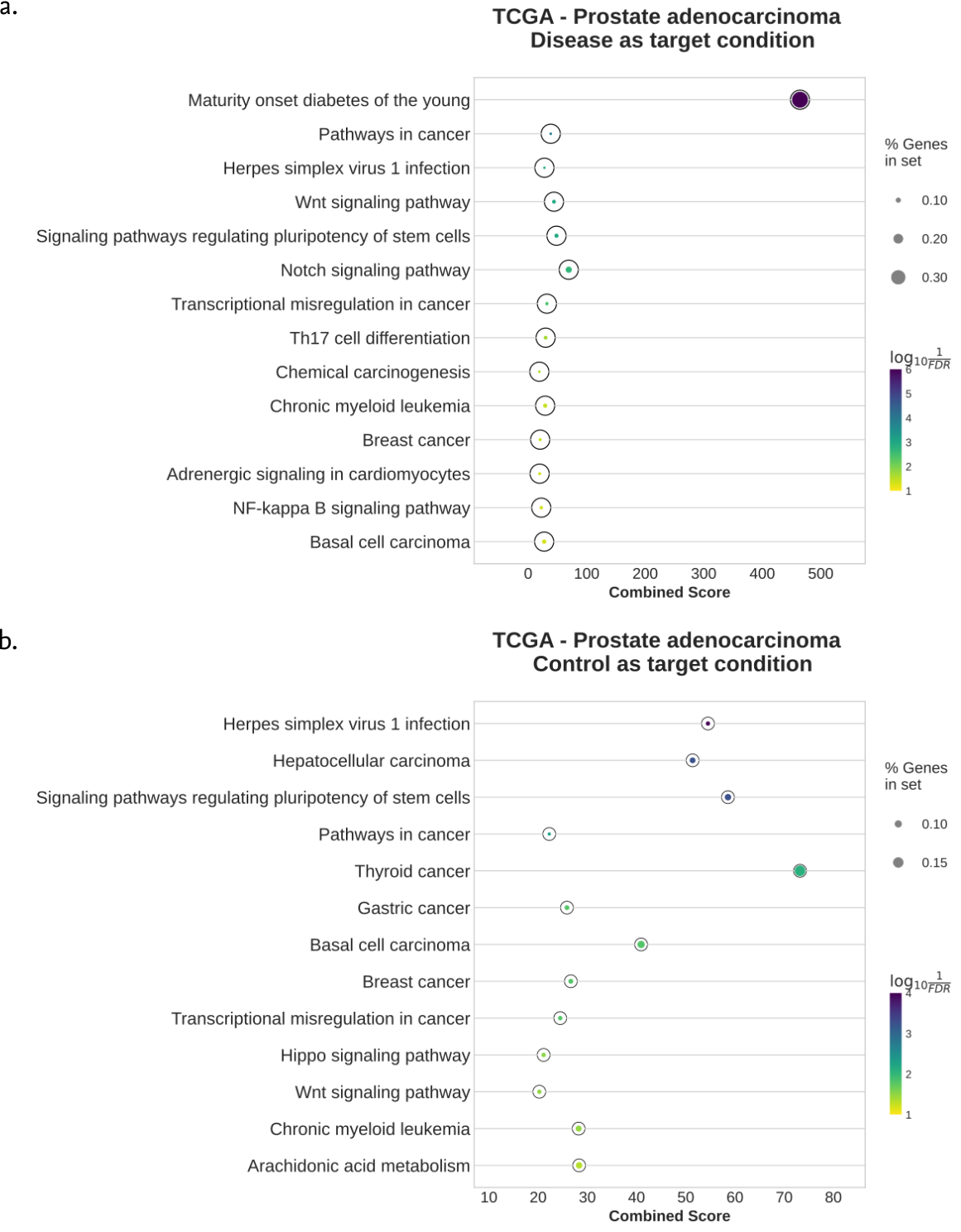

Supplement: vbae034_Supplementary_Data [file vbae034_supplementary_data.zip › FigS24_prad_target_conditions.png]

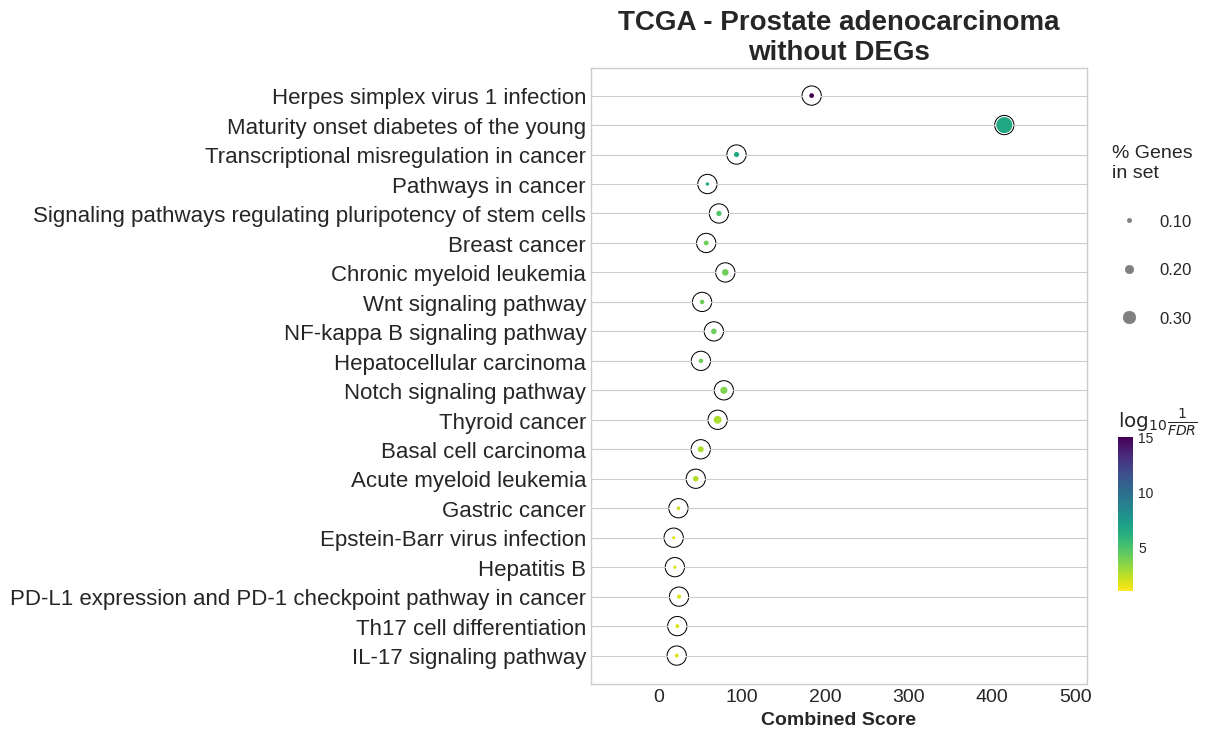

Supplement: vbae034_Supplementary_Data [file vbae034_supplementary_data.zip › FigS25_TCGA_PRAD_without_degs.png]

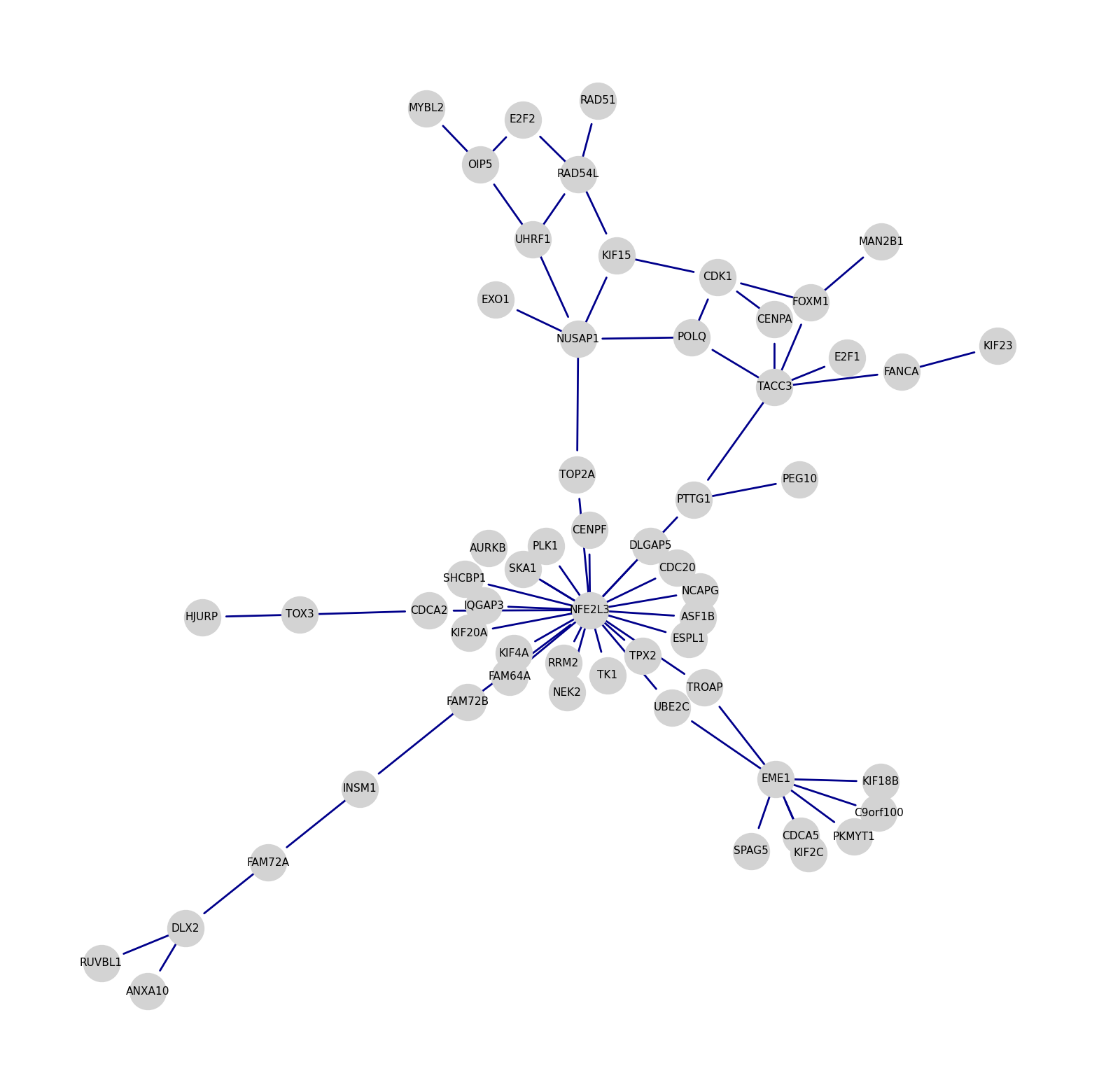

Supplement: vbae034_Supplementary_Data [file vbae034_supplementary_data.zip › FigS26_prad_diffcoex_module.png]

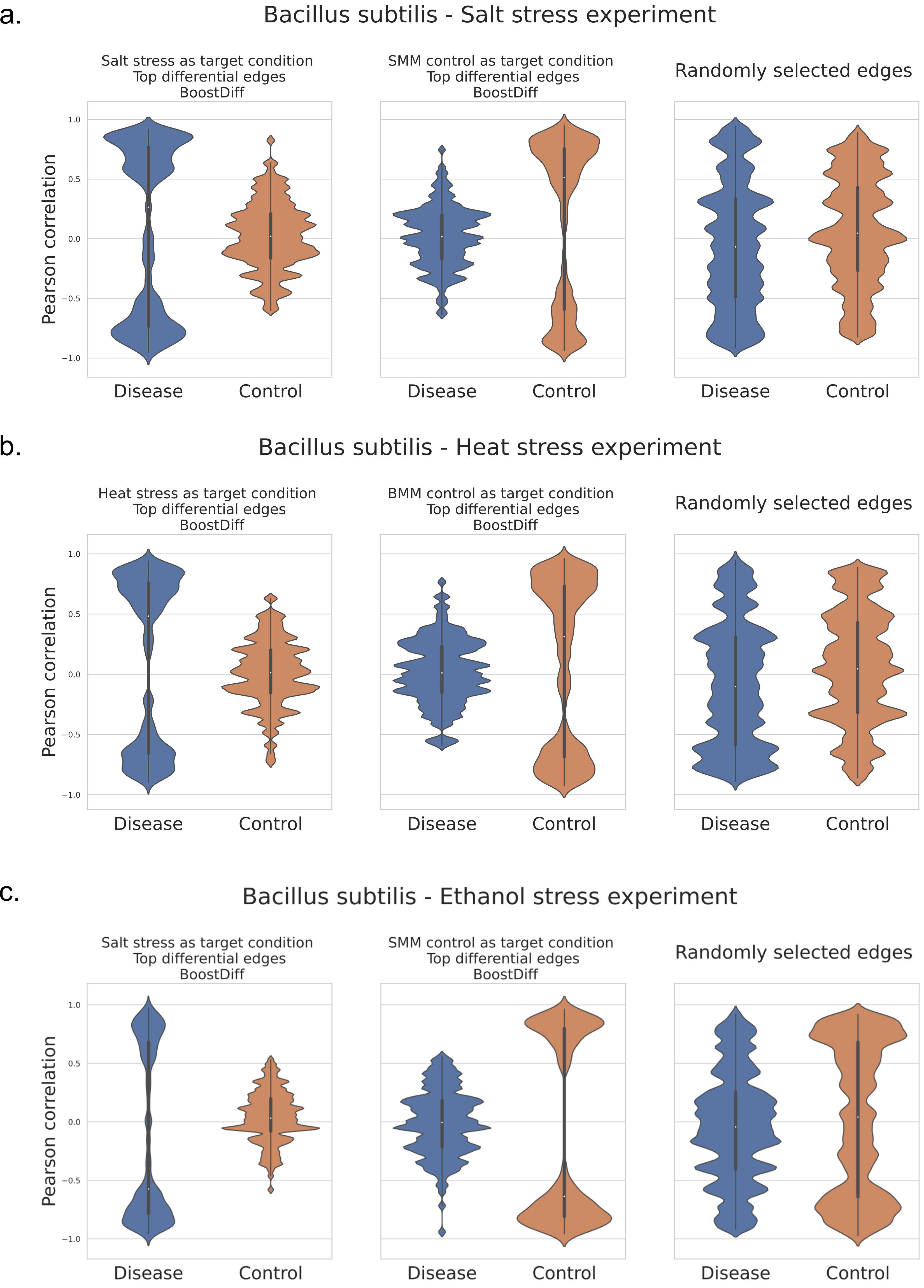

Supplement: vbae034_Supplementary_Data [file vbae034_supplementary_data.zip › FigS27_correlation_analysis_bsubt_smote_salt_and_heat_stress.png]

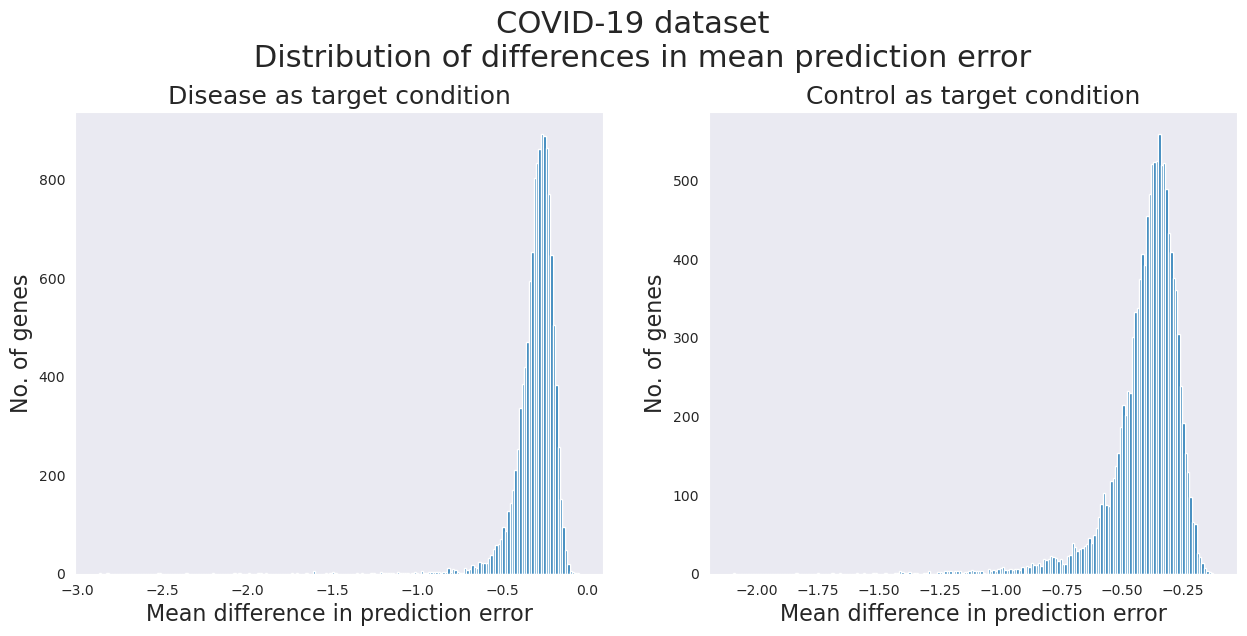

Supplement: vbae034_Supplementary_Data [file vbae034_supplementary_data.zip › FigS3_covid19_distributions.png]

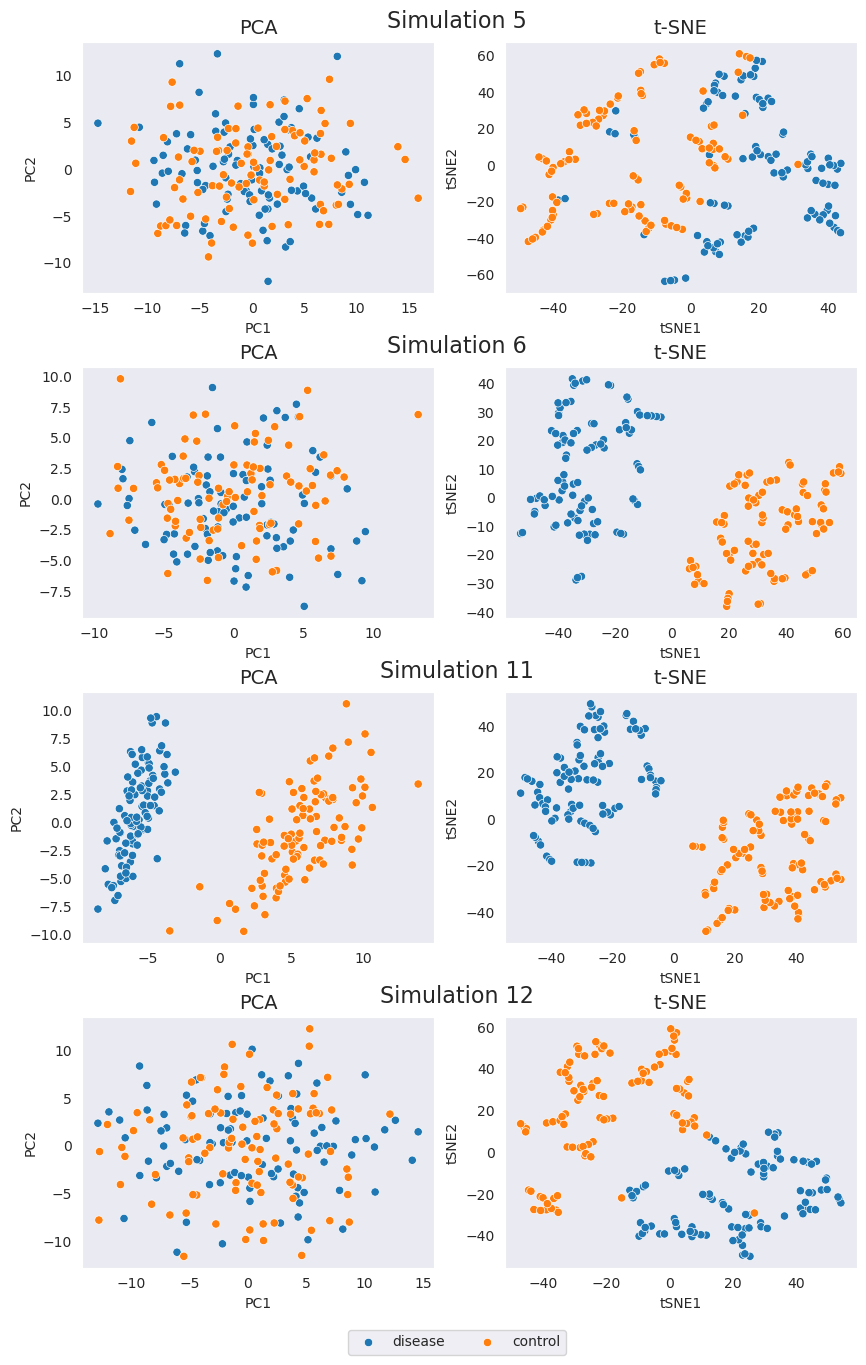

Supplement: vbae034_Supplementary_Data [file vbae034_supplementary_data.zip › FigS4_PCA_vs_tSNE.png]

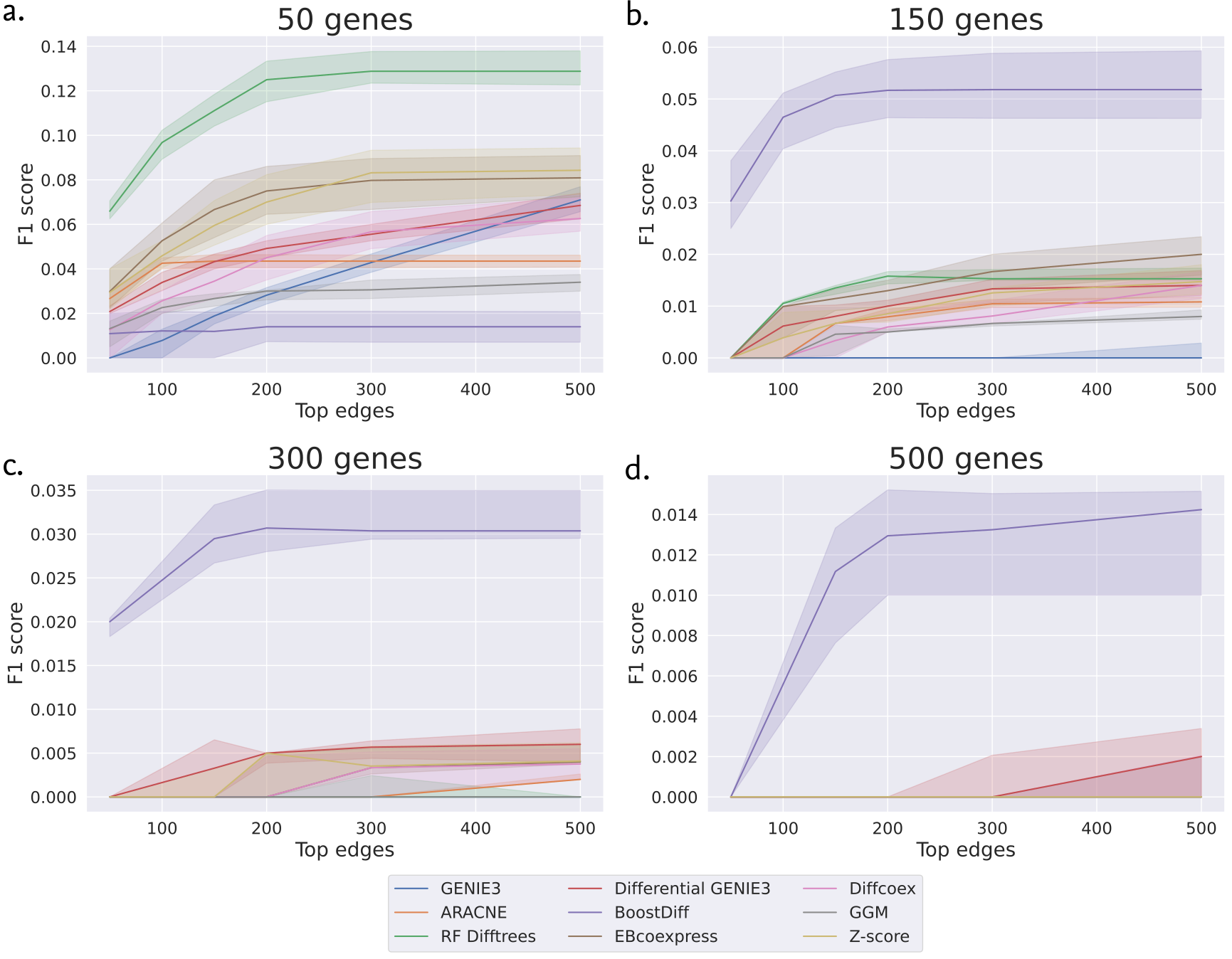

Supplement: vbae034_Supplementary_Data [file vbae034_supplementary_data.zip › FigS5_evaluation_varying_top_edges.png]

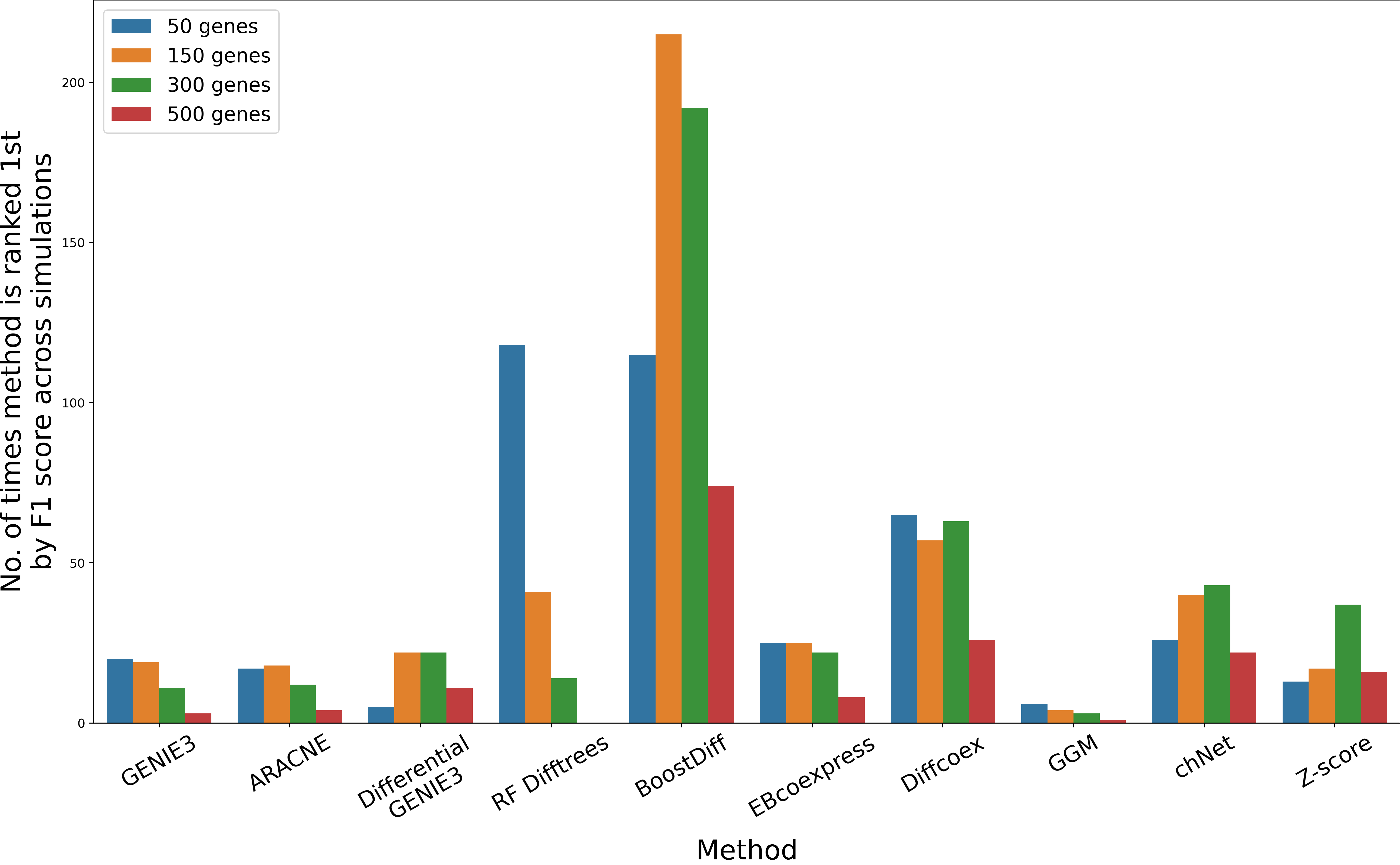

Supplement: vbae034_Supplementary_Data [file vbae034_supplementary_data.zip › FigS6_ranks_f1score.png]

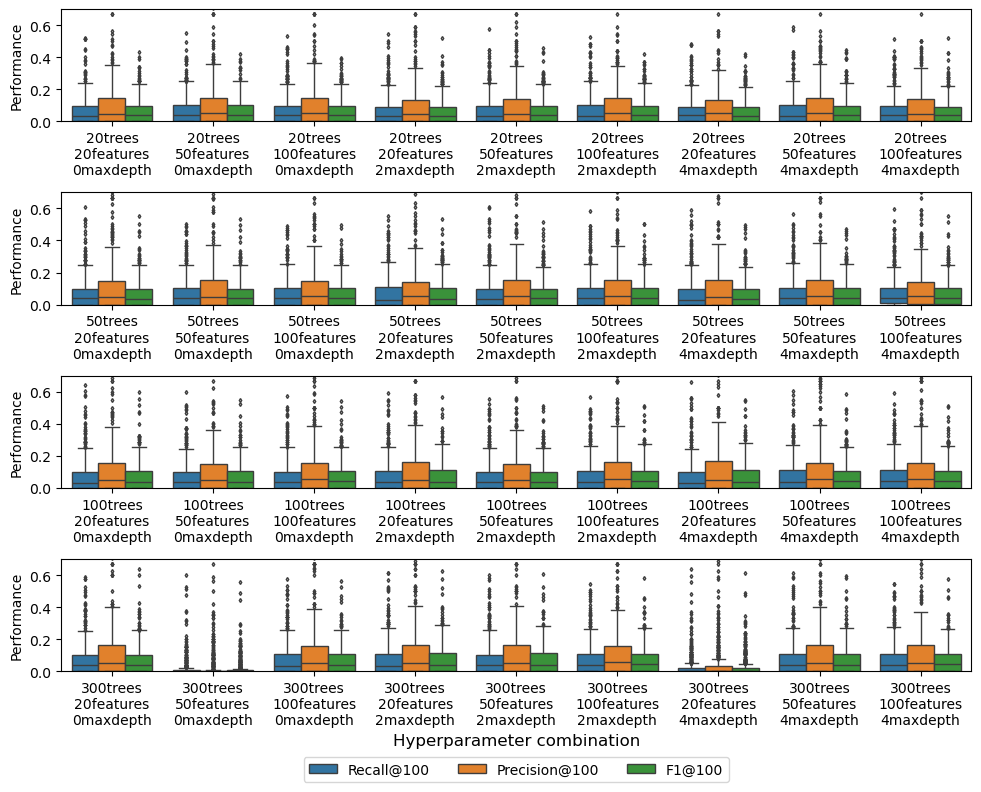

Supplement: vbae034_Supplementary_Data [file vbae034_supplementary_data.zip › FigS7_hyperparameter_combinations.png]

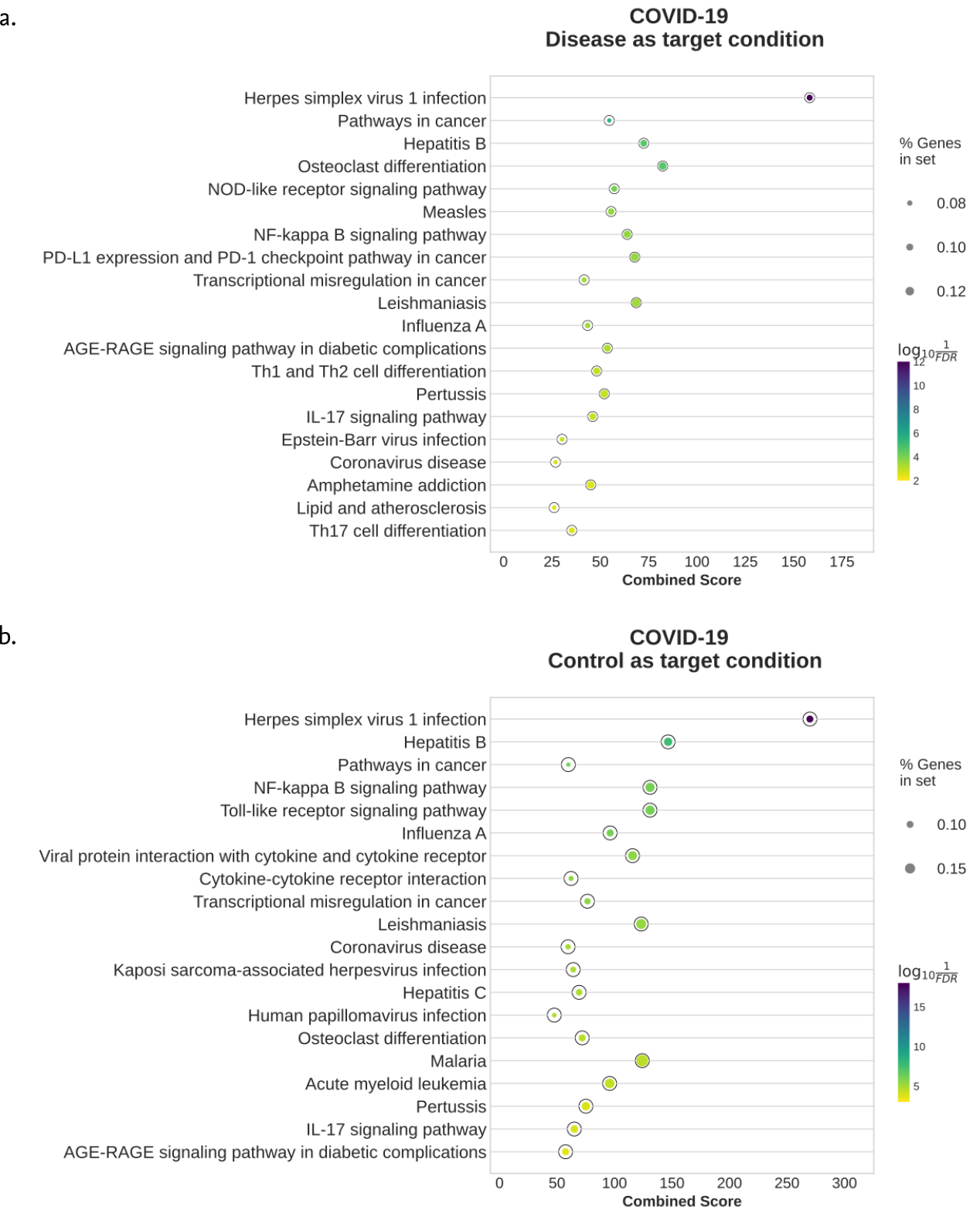

Supplement: vbae034_Supplementary_Data [file vbae034_supplementary_data.zip › FigS8_covid_target_conditions.png]

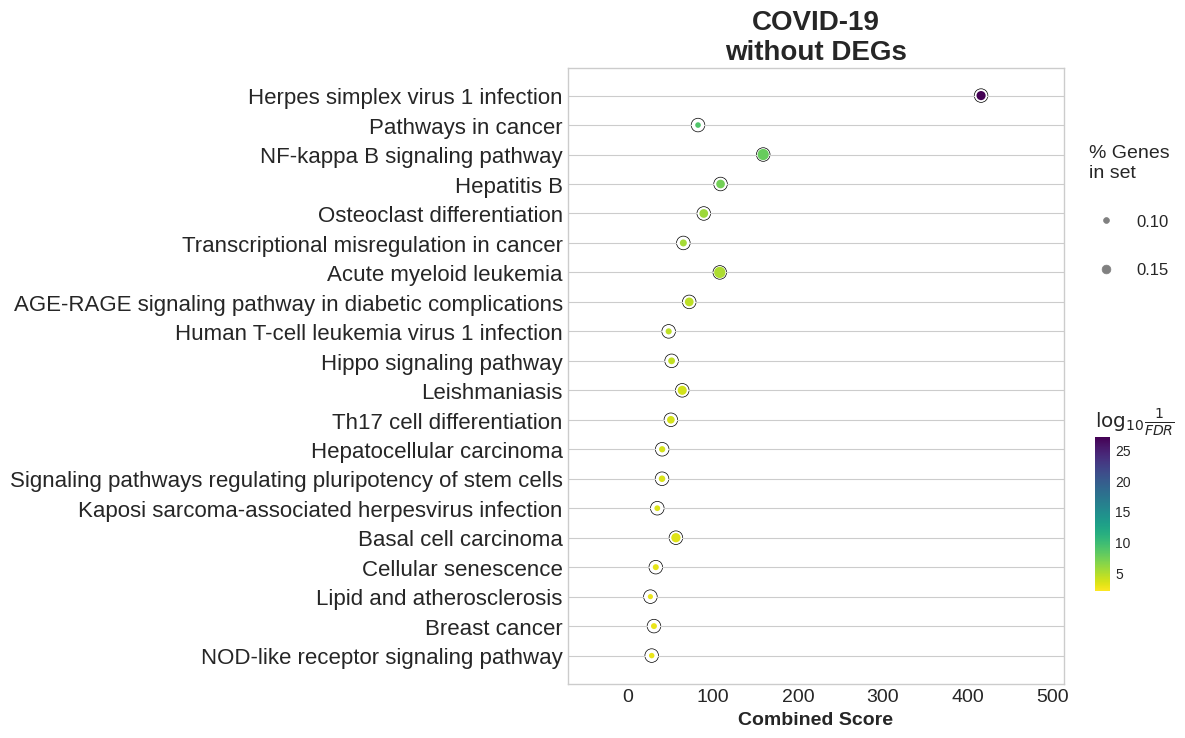

Supplement: vbae034_Supplementary_Data [file vbae034_supplementary_data.zip › FigS9_covid19_without_degs.png]
